# Unveiling the physical properties of AlMgX_3_ (X = Cl and I) for emerging energy applications

**DOI:** 10.1039/d6ra03627a

**Published:** 2026-09-15

**Authors:** Hamza Zafar, Mubashir Hussain, Farooq Ali, Wajeehah Shahid, Soumaya Gouadria, Hamid Ullah, Zeshan Aslam Khan

**Affiliations:** a Department of Physics, University of Lahore Lahore Campus Pakistan; b Government MC Model High School, Department of Education Chichawatni Sahiwal 57200 Punjab Pakistan; c Punjab Group of Colleges Airline Campus, 471 Lahore Pakistan; d Department of Physics, College of Science, Princess Nourah bint Abdulrahman University P.O. Box 84428 Riyadh 11671 Saudi Arabia; e Department of Physics, RIPHAH International University Lahore Campus Pakistan hamid.uou@gmail.com; f Department of Mathematics, Saveetha School of Engineering, SIMATS, Saveetha University Chennai 602105 Tamil Nadu India; g Multiscale Materials Modeling Laboratory, Department of Physics, University of Ulsan Ulsan 44610 Republic of Korea; h International Graduate School of Artificial Intelligence, National Yunlin University of Science and Technology 123 University Road, Section 3 Douliou Yunlin 64002 Taiwan, R.O.C

## Abstract

In this study, we employed first-principles density functional theory (DFT) to investigate the structural, elastic, optoelectronic, and thermoelectric properties of lead-free halide perovskites, AlMgX_3_ (X = Cl and I), for renewable energy applications. AlMgX_3_ exhibit an energetically favourable nature due to their lower formation energy (−4.17 eV per f.u.). Additionally, AlMgX_3_ are mechanically favourable with high elastic constants. We found that AlMgI_3_ exhibits higher ductility (*B*/*G* = 2.97) and anisotropy. Furthermore, AlMgX_3_ remain stable at room temperature, as confirmed by AIMD simulations. Both AlMgX_3_ exhibit a semiconducting nature. Interestingly, a direct band gap (3.60 eV) is observed for AlMgCl_3_, which could be promising for optical devices. Moreover, AlMgI_3_ demonstrates superior absorption in the visible spectrum, attributed to its reduced band gap, and exhibits a higher static dielectric constant, which could be beneficial for photovoltaic applications. Furthermore, AlMgI_3_ achieves a higher figure of merit (*ZT* = 0.61 at 300 K) due to enhanced electrical conductivity and power factor, though its indirect gap limits its high-temperature performance compared to AlMgCl_3_ (*ZT* = 0.70 at 800 K). Moreover, the suitable band edges make them promising for water splitting. These results underscore the suitability of AlMgX_3_ for a wide range of applications such as solar cells, optoelectronics, thermoelectrics and photocatalysis.

## Abstract 2

The AlMgX_3_ exhibits energetically favourable nature due lower formation energy (−4.17 eV/f.u.). AlMgX_3_ remain stable at room temperature. Direct band gap is observed for AlMgCl_3_, which could be promising for the optical devices.

## Graphical abstract

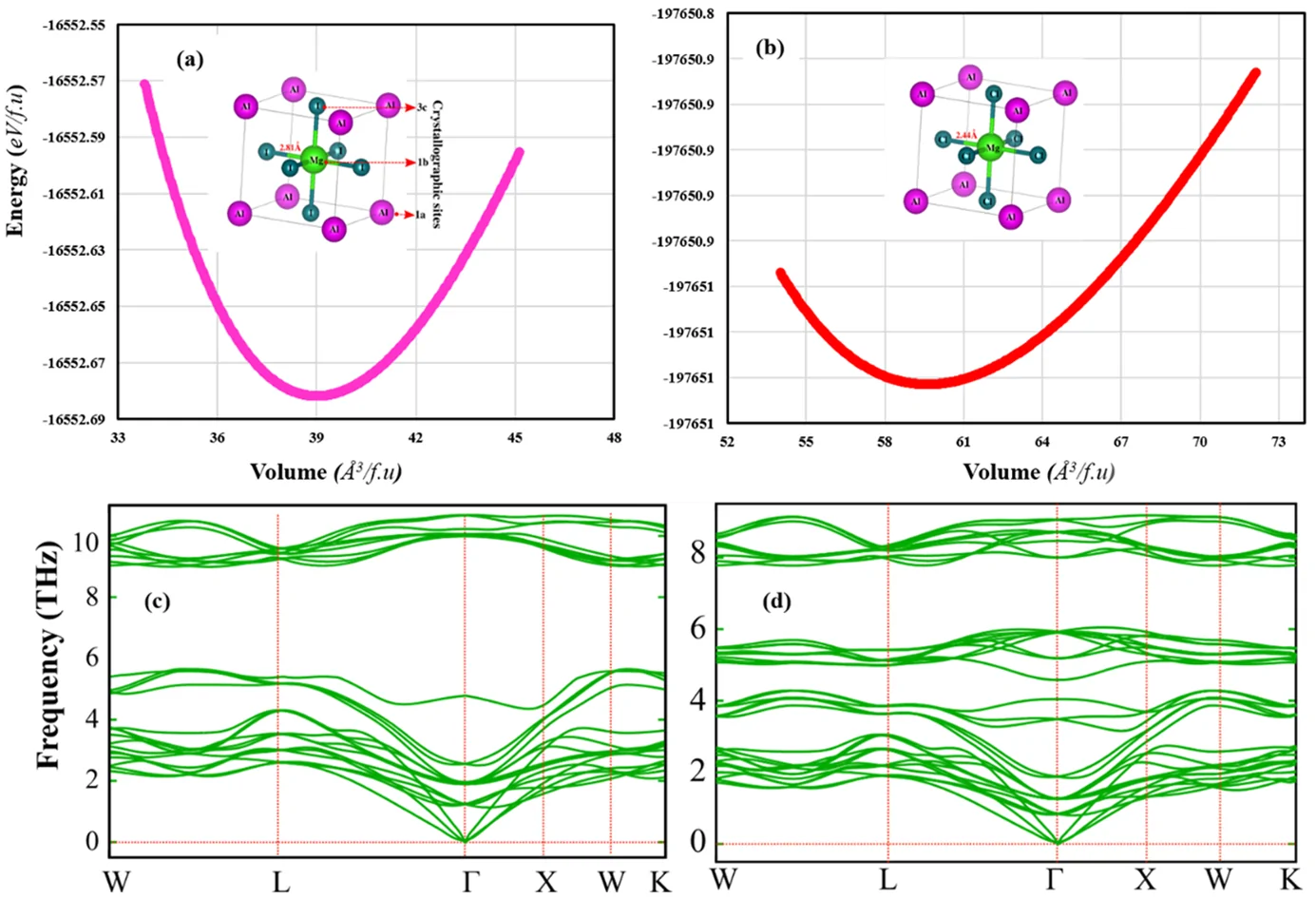

## 1 Introduction

Meeting the rising energy demands of modern society is one of the major concerns of the 21st century.^1^ Recent studies on global energy resources indicate that basic fossil fuels, including natural gas, oil and coal, are expected to be consumed within this century, prompting an important drive to advance renewable energy supplies and feedstocks. In contrast to the finite supply of fossil fuels, the sun serves as an optimal energy source due to the infinite potential of solar energy, which is widely accessible globally.^2^ In this regard, solar cell technology has significantly progressed in recent decades due to the increasing worldwide need for renewable energy.^3^ Solar cell technology distinguishes itself from other green energy sources owing to its economical processing and superior performance. Any material exhibiting effectiveness in power generation is subjected to thorough examination for its possible use in solar cell manufacturing. The inherent properties of the material plays a crucial role in solar cell manufacturing. Ongoing scientific research is essential to create a solar cell that is cost-effective, reliable and efficient.

Research has been conducted on the influence of humidity on the efficacy of economical perovskite photovoltaic (PV) devices.^4^ The drawbacks of Pb-based perovskites, such as instability, toxicity and inadequate mechanical properties, which impede their further use, have been thoroughly examined.^5^ Perovskites have a unique property that allows the effective absorption of visible light, suggesting their prospective use in PV devices in the future. Currently, the metal halide perovskite family, particularly the non-toxic AlMgX_3_ family, has garnered significant interest in the green solar cell sector owing to its remarkable structural, optoelectronic, and mechanical characteristics. The integration of compound characteristics for applications such as optoelectronics, quantum computing, biomedical electronics and power conversion is becoming common in modern semiconductor device applications.^6–11^ Various investigations have been carried out to improve the efficacy of these devices for specific applications by reducing inefficiencies and augmenting their performance and longevity. The mechanical and magnetic characteristics have been investigated to develop more dependable and economical PV and photocatalytic systems.^12,13^

Moreover, ABX_3_ materials are favourable due to their low fabrication cost and ecological sustainability. Halide-based perovskites are very prominent owing to their several physical characteristics, including low formation energy, indirect bandgap and a nonvisible region in the optical spectrum.^14–16^ Furthermore, they have exceptional thermal efficiency. Moreover, perovskite-based semiconductors have considerable potential for use in solar cells, photodetectors, electronic devices and LEDs.^17^ Perovskite halides are important in the thermoelectric field, such as thermoelectric generators, thermocouples and thermocoolers, due to their outstanding thermal efficiency.

The efficiency of perovskite-based PV cells has greatly enhanced over time, reaching an impressive 23.2% in 2019, up from 3.8% in 2009. The maximum energy conversion efficiency reported for lead-based perovskite PV cells is 25.7%,^18^ although their lifespan is diminished owing to variables such as UV radiation, humidity, and temperature.^19^ At first, Pb-based organic perovskite PV cells (CH_3_NH_3_PbI_3_) were created,^20–22^ but their use was limited due to their hazardous and unfavourable properties.^23^ To minimize their toxic nature, eco-friendly cations such as Mg^2+^ and Ca^2+^ have been used to substitute Pb^2+^ cations, while Al^3+^ and Ga^3+^ cations have substituted the organic equivalents. As a result, a new configuration, ABX_3_, has been achieved, in which A and B denote divalent and trivalent cations, respectively, and X represents an atom from the halogen group.

According to the Shockley–Queisser theory, perovskites have the ability to achieve an energy conversion efficiency of 33% if the value of the energy gap is maintained between 1.2 and 1.4 eV.^24^ Two primary conditions must be fulfilled to enhance the efficacy of energy cells and solar systems using perovskite halides. First, a narrow band gap ranging from 0.8 to 2.2 eV (ref. 25) is required because optimal energy conversion efficiencies^26^ are shown by effective light collectors with electronic band gaps between 1.3 and 1.7 eV. Second, a direct energy band gap is crucial for enhancing the energy conversion efficiency of PV absorbers.^27^

The literature strongly suggests a close relationship between physical properties and the introduction of different halogen atoms in perovskite compounds. Mera *et al.* studied the optical, electronic and thermoelectric properties of Cs_2_CuSbX_6_.^28^ It was found that the band gap could be engineered from 1.04 eV to 0.46 eV by replacing the X atom with Cl, Br, and I, suggesting that Cs_2_CuSbX_6_ is the best candidate for optoelectronic and solar cell devices. Shen *et al.*^29^ determined the indirect and direct nature of electronic band gaps of Pb-free double perovskites Cs_2_AgInCl_6_ and Cs_2_AgSbCl_6_ by substituting In and Sb for Bi, respectively. The same study disclosed the photoactive uses of perovskites. M. I. Kholil *et al.*^30^ examined the effects of Tc and Mo doping on the electronic behaviour of the Pb-free perovskite CsSnBr_3_ employed in optoelectronic applications.^30^ They also studied the differences in the visible light absorption and band gap tunability of Pb halide (CsPbCl_3_) and Pb-free (CsSnCl_3_) perovskites for optoelectronic applications.^31^ In 2017, Roknuzzaman *et al.* reported that the use of CsBX_3_ (X = I, Br, Cl; B = Ge, Sn) significantly surpasses that of the Pb-based halide perovskites CsPbX_3_ (X = Br, Cl, I) in optical and electronic applications, particularly in PV cells.^32^ Recently, Fatima *et al.* also explored the structural and optoelectronic properties of AgYF_3_ (Y = Mg, Sr) using first-principles calculations and showed the potential of the studied materials in PV energy harvesting.^33^ Al-based halide and fluoro perovskites have recently attracted growing research interest. Among these, AlMF_3_ (M = Ca, Cd) are identified to exhibit direct band gaps with potential for optoelectronic applications.^34^ Similarly, a recent DFT-based investigation on AlMF_3_ (M = Ca, Zn, Ge) confirmed their potential for photovoltaic applications, based on their electronic characteristics.^35^ Moreover, the first-principles analysis of AlGeX_3_ perovskites (X = F, Cl, Br, I) demonstrated halogen-dependent band transition from an indirect to direct band gap and optical tunability for optoelectronic and photovoltaic applications.^36^ Furthermore, Ghani *et al.* explored the role of uniaxial strain in modulating the functional properties of AlXF_3_ (X = Ge, Sn) for optoelectronic applications.^37^

To the best of our knowledge, no prior experimental study has investigated the physical properties of AlMgX_3_ (X = Cl and I) halide perovskites. Previous Al-based perovskite studies have predominantly focused on fluoride (F) systems or alternative B-site cations (Ge, Cr, B, Ca, and Cd), and the substitution of Cl and I introduces a fundamentally different ionic radius, electronegativity, and polarizing environment, leading to distinct electronic structures, band gap characters, and optical response profiles that cannot be extrapolated from fluoride-based analogues. Other halide-based perovskites, AlMgF_3_ (ref. 38) and AlMgBr_3_,^39^ which are closely related to our studied material, AlMgX_3_ (X = Cl and I), have also been investigated due to their high machinability index and solar cell performance. Due to the very close family relation of AlMgX_3_ (X = Cl and I) with AlMgF_3_ and AlMgBr_3_, they may also have potential in thermoelectric and solar cell applications. The present work provides a comprehensive multi-property investigation encompassing structural stability and electronic, optical, mechanical, thermoelectric, and photocatalytic properties of AlMgCl_3_ and AlMgI_3_ for energy applications.

## 2 Computational methodology

The present study was carried out using the Wien2k code based on density functional theory.^40–42^ In order to compute the lattice constant and energy of the studied materials in the ground state, the cubic structures (*Pm*3̄*m*) of AlMgX_3_ (X = Cl and I) were optimized by employing the PBEsol-GGA potential. The relaxed crystal geometry was constructed using VESTA software.^43^ The physical features, such as elastic, electronic, optical and thermoelectric features, were computed using the same lattice parameters that were obtained after structure optimization. The finite strain method was employed to determine the elastic moduli as well as elastic constants embodied in the Wien2k code.^44^ The ELATE tools^45^ were utilized to generate 3D anisotropic plots for the shear modulus, Young's modulus and Poisson's ratio. The TB-mBJ^46,47^ potential-based self-consistent field (SCF) converged the entire energy of the systems with proper energy and charge convergence criteria of 0.000001 and 0.000001, respectively. The TB-mBJ potential is considered comparatively better than GGA^48^ and PBEsol^49^ techniques. Furthermore, to resolve the charge neutrality issue, we performed a Bader charge analysis^50,51^ on the relaxed, self-consistent charge density of AlMgCl_3_ and AlMgI_3_. The computed atomic charges deviated substantially from the formal ionic values of +3 (Al), +2 (Mg) and −1 (X).

The electronic systems within the muffin-tin (MT) sphere were predicted to describe the spherical harmonics, but in the interstitial area, they typically resembled plane waves. The electronic states (3p^1^, 3s^2^), (3s^2^), (3s^2^, 3p^5^) and (5s^2^, 5p^5^) were considered as core states of Al, Mg, Cl and I elements, respectively. The product of the MT radius and maximum wave vector was set as *R*_MT_ × *K*_max_ = 7, the Gaussian parameter was *G*_max_ = 16, and the angular momentum at *l*_max_ was 10 in the reciprocal lattice to adjust the starting setting. To confirm the dynamical stability, we computed the phonon spectra.^52^ A large supercell of 6 × 6 × 6 was used for phonon calculations using the finite difference method to compute the phonon spectra using the phonopy package^52^ with VASP^53^ as the calculator. Additionally, to verify the thermodynamic stability of AlMgX_3_ (X = Cl and I), we performed *ab initio* finite-temperature molecular dynamics (MD) simulations at 300 K for 5000 steps with a time step of 1 fs using the Nose–Hoover heat bath scheme.^54,55^ A fine grid in the Brillouin zone was essential for *k*-space incorporation in optical and electronic calculations. On the basis of this knowledge, we used 15 × 15 × 15 and 23 × 23 × 23 Monkhorst–Pack grids to determine electronic and optical properties, respectively. The Kramers–Kronig^56^ relation was used to determine the dielectric properties of AlMgX_3_ (X = Cl and I). To determine the thermoelectric characteristics, electronic structures computed using the TB-mBJ potential were put into the BoltzTraP code.^57^ The temperature was maintained between 50 K and 800 K, with the relaxation time set at *τ* = 10^−14^. Furthermore, we followed ref. 58, where the thermoelectric transport coefficients were calculated using the semiclassical Boltzmann transport theory^57^ within the constant relaxation time approximation (CRTA), as implemented in the BoltzTraP code,^57^ for the computation of the thermoelectric parameters of AlMgX_3_ (X = Cl and I). We also calculated the lattice thermal conductivities (*κl*) of AlMgX_3_ (X = Cl and I) by employing the Phono3py^59^ code.

## 3 Results and discussion

### 3.1 Structure and stability of AlMgX_3_ (X = Cl and I)

**Fig. 1:**
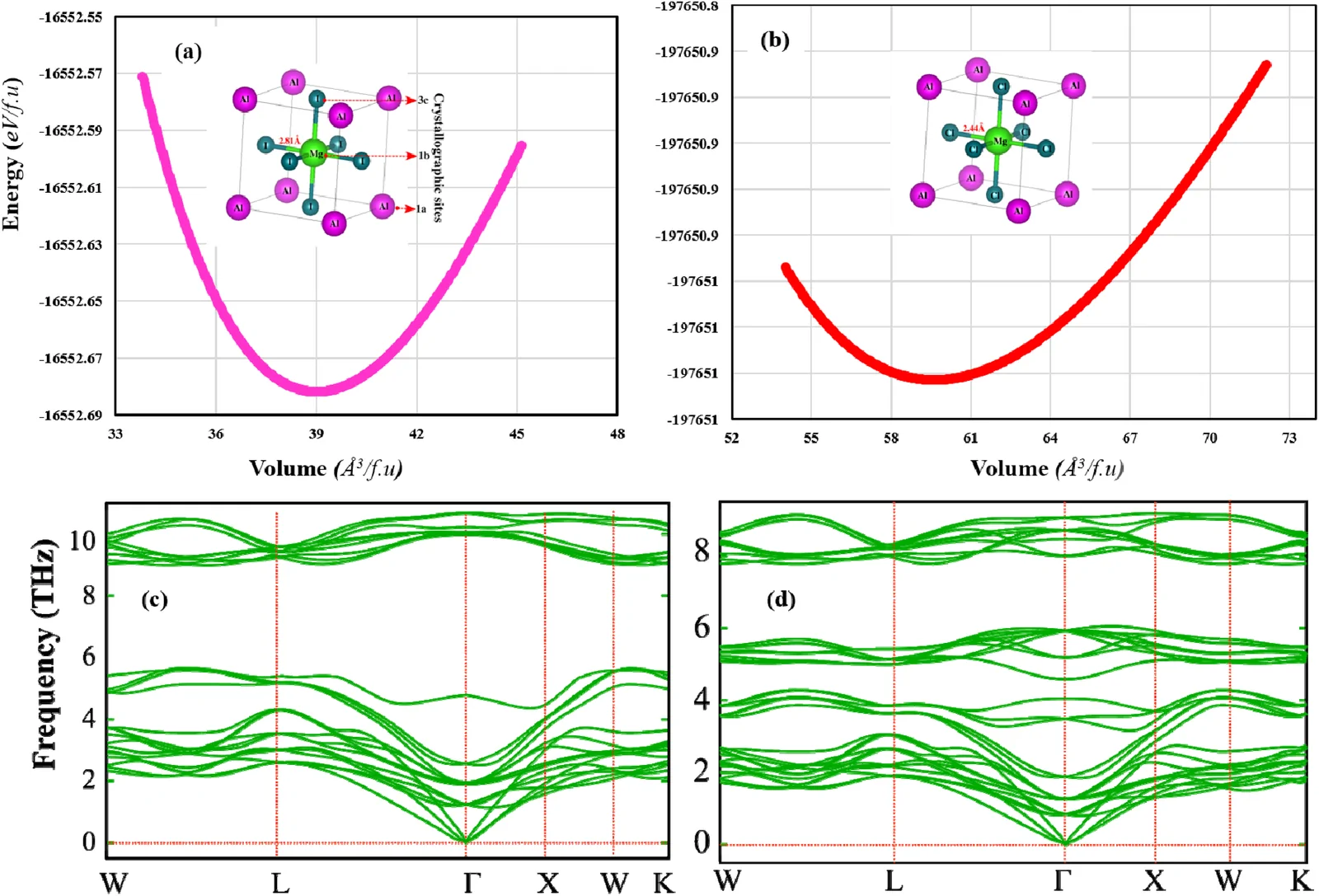
Optimized energy *vs.* volume and the phonon spectra of (a and c) AlMgCl_3_ and (b and d) AlMgI_3_. The insets show the relaxed structures of the respective materials.

We conducted a structural study of AlMgX_3_ by improving their atomic arrangement and correlating the findings with the well-known Birch–Murnaghan equation. Both compounds, AlMgCl_3_ and AlMgI_3_, have a similar space group, *Pm*3̄*m*, and their relaxed atomic structures are presented in Fig. 1. The insets of Fig. 1(a and b) illustrate the coordination of Al and Mg at (0, 0, 0) and (0.5, 0.5, 0.5), respectively, and the coordinates of the three X atoms occupy the positions (0.5, 0.5, 0), (0.5, 0, 0.5) and (0, 0.5, 0.5).^38,60^ To compute the lattice constants in the ground state, the optimized volumes of each of the lead-free perovskites were calculated before assessing their physical properties. The curves of the optimized volume reveal the equilibrium states of AlMgI_3_ with a volume of 59.63 Å^3^ per f.u. and AlMgCl_3_ with a volume of 39.01 Å^3^ per f.u. in the ground state, as depicted in Fig. 1. After calculating the optimized volume, we determined the lattice constants of AlMgCl_3_ and AlMgI_3_, which are 4.89 Å and 5.63 Å, respectively. The obtained values of lattice constants in the relaxed form for AlMgCl_3_ and AlMgI_3_ are comparable with the reported theoretical data of AlMgBr_3_ (*a* = 5.28 Å)^39^ and AlMgF_3_ (*a* = 4.14 Å),^61^ as well as experimentally studied data of CsSnBr_3_ (*a* = 5.80 Å).^62^ Moreover, we determined the formation energy of AlMgX_3_ to check their relative stability. We used the relation *E*_f_ = [*E*_AlMgX_3__ − *n*{*E*_Al_ + *E*_Mg_ + *E*_X_}]/*N*,^41^ where *E*_AlMgX_3__, *E*_Al_, *E*_Mg_, and *E*_X_ are the total energy of AlMgX_3_ (X = Cl and I) per unit cell and the energies of the isolated Al, Mg, and X atoms, respectively, *n* is the number of atoms of each element in the unit cell, and *N* = 3 is the number of atoms in the unit cell. The values of the *E*_f_ are estimated to be −2.98 eV per f.u. and −4.17 eV per f.u. for AlMgCl_3_ and AlMgI_3_, respectively. Our findings on the formation energy contrast with recently reported research data, such as Rb_2_Ca_2_Cd_3_O_10_ (−4.36 eV per f.u.),^41^ CsCaCl_3_ (−3.96 eV)^40^ and CsCaBr_3_ (−3.54 eV).^40^ Thus, our evaluated *E*_f_ values demonstrate the stable structure of AlMgX_3_ (X = Cl and I) and can be easily incorporated into experiments. Compared to AlMgCl_3_, AlMgI_3_ displays a lower formation energy. Thus, AlMgI_3_ could be more favourable for experiments. Additionally, we performed Bader charge analysis^50,51^ to confirm the charge neutrality of AlMgX_3_. This method partitions the total electron density into non-overlapping atomic basins separated by zero-flux surfaces, where ∇*ρ*(*r*)·*n* = 0, allowing quantification of the net charge localized on each atom independent of the choice of the basis set or orbital population scheme. Bader charge analysis shows that the effective charges on Al, Mg and X deviate markedly from the formal ionic values (Al^3+^, Mg^2+^, and X^−^). For AlMgCl_3_, the computed charges are *q*(Al) = +0.85 e, *q*(Mg) = +1.55 e and *q*(Cl) = −0.80 e per Cl atom; for AlMgI_3_, they are *Q*_Al_ (+0.70 e), *Q*_Mg_ (+1.44 e), and *Q*_I_ (−0.72 e) per atom, as tabulated in Table 1. In both compounds, the sum of the charges is zero, within numerical accuracy [0.85 + 1.55 + 3(−0.80) = 0 for AlMgCl_3_; 0.70 + 1.44 + 3(−0.72) = −0.02 for AlMgI_3_], confirming overall cell neutrality. Critically, the individual charges deviate substantially from the nominal ionic values of +3 (Al), +2 (Mg) and −1 (X), and the effective charge on Al is only ≈28–23% of its formal ionic value, while that on Mg is only ≈78–72%, indicating pronounced covalent charge-transfer suppression and significant orbital hybridisation in the Al–X and Mg–X bonds. This demonstrates that AlMgX_3_ are not classical, fully ionic ABX_3_ perovskites, but a covalency-stabilised framework whose real, DFT-derived charge distribution—rather than a formal oxidation-state count—governs their structural stability.

**Table 1:** Evaluated lattice constants (*a*), optimized volumes (*V*), bond lengths (Mg–X), formation energies (*E*_f_), band gaps (*E*_g_), static dielectric constants (*ε*_1_(0)), figures of merit (*ZT*), and Bader charges (*Q*) of AlMgCl_3_ and AlMgI_3_

	AlMgCl_3_	AlMgI_3_	Ref.
*a* (Å)	4.89	5.63	4.14,^61^ 5.80 (ref. 62)
*V* (Å^3^ per f.u.)	39.01	59.63	—
Mg–X (Å)	2.44	2.81	2.84–3.14 (ref. 63)
*E*_f_ (eV per f.u.)	−2.98	−4.17	−4.36,^41^ −3.54 (ref. 40–42)
*E*_g_ (eV)	3.60	2.44	2.92–3.45 eV (ref. 64)
*ε*_1_(0)	3.55	5.14	3.27 (ref. 39)
*ZT*	0.70	0.68	0.76 (ref. 65)
*Q*_Al_ (e)	0.85	0.70	—
*Q*_Mg_ (e)	1.55	1.44	—
*Q*_Cl_ (e)	−0.80	—	—
*Q*_I_ (e)	—	−0.72	—

Additionally, we confirmed dynamical stability using phonon spectra. The absence of imaginary frequency modes across the entire Brillouin zone indicates that both materials are dynamically stable, as depicted in Fig. 1.

**Fig. 2:**
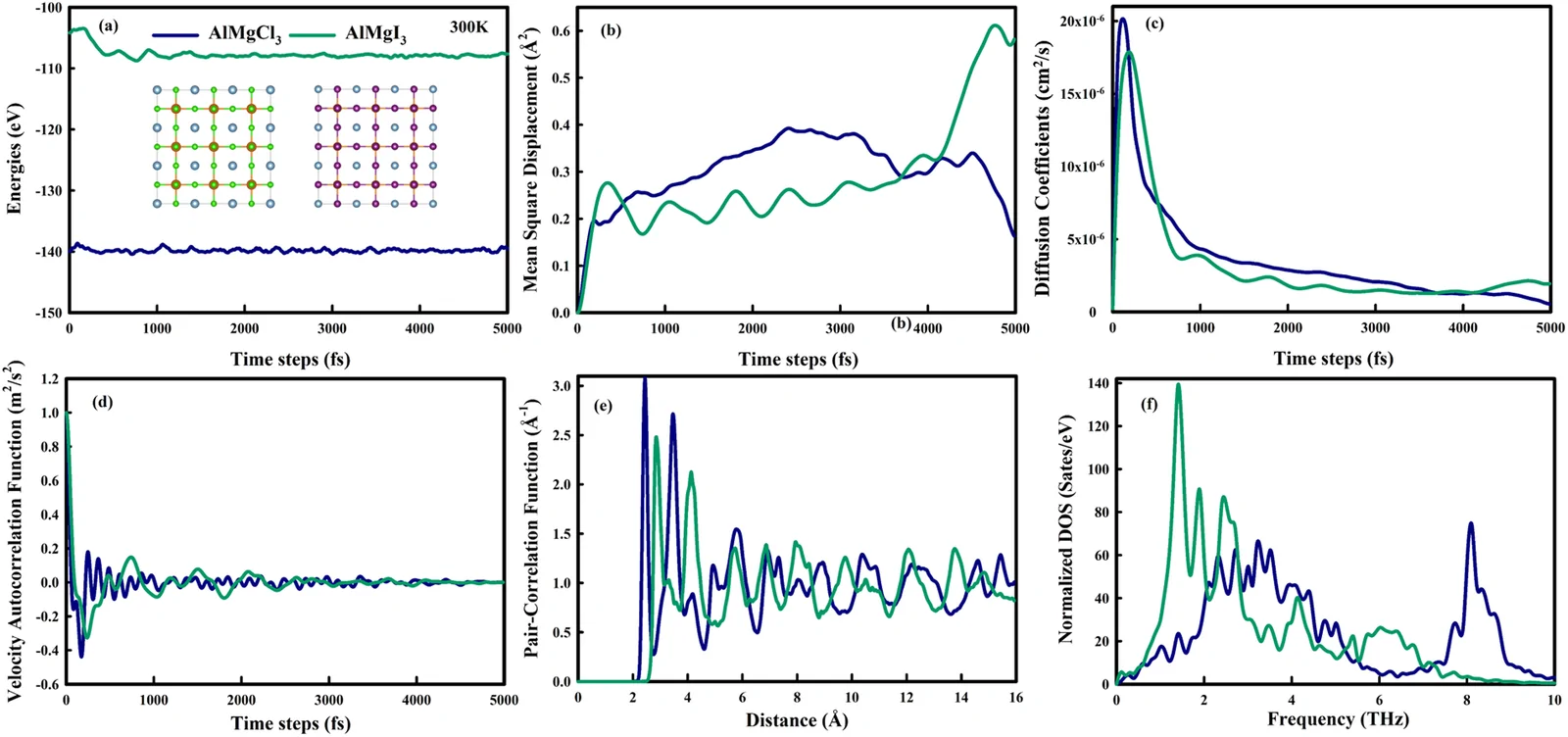
AIMD simulations: (a) total energies, (b) mean square displacement, (c) diffusion coefficients, and (d) velocity autocorrelation function with respect to time steps, (e) pair correlation function with respect to distance, and (f) the normalized DOS with respect to the frequency of AlMgI_3_.

We performed AIMD simulations to estimate the energy, mean square displacement (MSD), time-dependent diffusion coefficient (TDDC), velocity autocorrelation function (VACF), pair correlation function (PCF), and normalized phonon density of states (NPDOS), as illustrated in Fig. 2. It is observed that the fluctuation of the total energy as a function of simulation steps of 5000, where the average values of the total energy remain nearly constant during the simulation, as shown in Fig. 2(a), thus confirming that the atomic structures are well-sustained during the simulation process.

To evaluate the atomic diffusion behavior of AlMgX_3_, we calculated the MSD as a function of simulation time (presented in Fig. 2(b)). In the initial period (0–600 fs), both the compounds exhibit a sharp increase in MSD, corresponding to the ballistic regime where atoms move freely before experiencing significant interactions. In the intermediate time period (∼600–3000 fs), a gradual increase in the MSD is observed, with noticeable oscillations, particularly for AlMgCl_3_, indicating the vibrational motion of atoms within local potential minima and repeated collision events. Furthermore, at higher time scales (>3000 fs), a clear difference can be seen between AlMgCl_3_ and AlMgI_3_. AlMgCl_3_ demonstrates a continuous increase in MSD, reaching approximately 0.6 Å^2^ at 5000 fs, which significantly enhances atomic mobility and diffusive behavior (see Fig. 2(b)). In contrast, AlMgI_3_ tends to saturate, and a slight decrease is observed after a longer time (>3000 fs), suggesting that the atomic motion is restricted and may likely undergo structural stabilization due to stronger interatomic interactions. Overall, the contrary MSD trends underscore the variations in the dynamical properties of AlMgX_3_, where one possesses relatively higher diffusivity, while the other remains localized.

In order to provide more insights into the dynamical behavior of AlMgX_3_, we derived the TDDC (shown in Fig. 2(c)) from the MSD (Fig. 2(b)). In the beginning, AlMgX_3_ exhibit a sharp increase in the TDDC, ranging from ∼10^−5^ to 10^−6^ cm^2^ s^−1^, which is consistent with the ballistic regime in the MSD calculated in Fig. 2(b). This enhancement may arise from the initial uncorrelated motion of the atoms before collisions dominate the dynamics. Additionally, as time increases, the TDDC decreases for both AlMgCl_3_ and AlMgI_3_, suggesting the transition to stabilization, reflecting the crossover from ballistic to diffusive motion.

Overall, the combined analysis of the MSD and diffusion coefficient clearly demonstrates that AlMgCl_3_ possesses a higher long-time diffusivity, whereas AlMgI_3_ exhibits reduced mobility and increased localization. This consistency in AlMgX_3_ validates the reliability of the diffusion analysis and highlights its distinct transport properties.

Fig. 2(d) presents the velocity autocorrelation function (VACF) as a function of time, providing further insights into the atomic dynamics in relation to the MSD (Fig. 2(b)) and diffusion coefficients (Fig. 2(c)). At short time scales, both systems exhibit a strong positive peak, corresponding to the ballistic regime, followed by rapid decay and oscillations with negative regions due to velocity reversals and caging effects. AlMgCl_3_ shows more pronounced and persistent oscillations, indicating stronger backscattering and restricted atomic motion, consistent with the MSD saturation and the decrease in the diffusion coefficient. In contrast, AlMgI_3_ displays faster damping of oscillations, suggesting weaker velocity correlations and enhanced atomic mobility, in agreement with its higher MSD growth and sustained diffusion at longer times. At extended time scales, both AlMgX_3_ converge toward zero, confirming the loss of velocity memory and the onset of diffusive behavior.

The PCF obtained from MD simulations reveals pronounced structural ordering in AlMgX_3_, as shown in Fig. 2(e). The sharp first peak at ∼2–3 Å indicates a well-defined nearest-neighbor coordination shell, confirming strong short-range interactions. The subsequent damped oscillations correspond to higher coordination shells, reflecting intermediate-range order. At larger distances, the PCF approaches unity, indicating the loss of long-range correlations and a homogeneous phase. The comparative profiles of AlMgX_3_ suggest differences in local packing and interaction strength, with the blue curve exhibiting relatively higher structural ordering.

The normalized phonon density of states (DOS) reveals the distinct vibrational behavior of AlMgX_3_, as shown in Fig. 2(f). AlMgCl_3_ is dominated by low-frequency modes (∼1–2 THz), indicating softer, collective lattice vibrations. In contrast, AlMgI_3_ shows a broader spectrum with a pronounced high-frequency peak (∼8 THz), suggesting stronger bonding and higher structural rigidity. These differences in the spectral distribution reflect variations in interatomic interactions and dynamical properties. Note that our calculations are in good agreement with the available literature.^32,55,66^

### 3.2 Mechanical properties

The structural stability and mechanical properties of any material are determined by computing the elastic constants (*C*_*ij*_). *C*_*ij*_ offers dynamic insights into a crystal's response to external pressure. The elastic feature describes how a material deforms under stress and then returns to its original shape following the removal of the stress. It is necessary to know the structural stability, binding properties among neighbouring atomic planes, and anisotropic nature of any studied material. A cubic material is often described by three fundamental elastic constants, namely, *C*_11_, *C*_12_ and *C*_44_. Table 2 presents the calculated *C*_11_, *C*_12_ and *C*_44_ and the Cauchy pressure (*C*_12_ − *C*_44_) for AlMgCl_3_ and AlMgI_3_ under pressure. A cubic structure is analysed under three stability conditions. When *C*_11_ + 2*C*_12_ > 0, it satisfies the spinodal criteria; and when *C*_11_ − *C*_12_ > 0, it meets the Born criteria, while it falls under the shear criteria when *C*_44_ > 0. Because all computed elastic constants for AlMgCl_3_ and AlMgI_3_ compounds satisfy these stability conditions (*C*_44_ > 0, *C*_11_ − *C*_12_ > 0, and *C*_11_ + 2*C*_12_ > 0),^67^ both studied compounds have mechanical stability. Moreover, *C*_12_ − *C*_44_ can define the brittleness and ductility of a material. A negative or positive value for *C*_12_ − *C*_44_ shows the brittleness or ductility of the studied material, respectively.^68^ Consequently, both compounds are predicted to be ductile because they have positive *C*_12_ − *C*_44_ values. However, AlMgI_3_ is more ductile than AlMgCl_3_, as shown in Table 2.

**Table 2:** Evaluated elastic constants and elastic moduli (bulk modulus, shear modulus and Young's modulus), Pugh's ratio, Poisson's ratio and Zener anisotropy of AlMgCl_3_ and AlMgI_3_

Parameters	AlMgCl_3_	AlMgI_3_	CsCaX_3_ (X = F, Cl, Br)^69^
*C*_11_ (GPA)	76.74	50.27	41.61–90.33
*C*_12_ (GPA)	19.29	11.30	8.61–24.4
*C*_44_ (GPA)	13.00	4.13	7.95–22.99
*C*_12_ − *C*_44_	6.29	7.16	
*B* (GPA)	38.44	24.29	19.61–46.40
*G* (GPA)	17.97	8.15	10.70–26.56
*Y* (GPA)	46.64	22.00	27.17–66.92
*B*/*G*	2.13	2.97	1.83–1.74
*ν*	0.29	0.34	0.27–0.26
*A*	0.45	0.21	0.48–0.69

Various important elastic characteristics, such as bulk (*B*), shear (*G*) and Young's (*Y*) moduli, Pugh's ratio (*B*/*G*), Poisson's ratio (*ν*), and Zener's anisotropic ratio (*A*), were computed on the basis of *C*_*ij*_. The *B* defines a material's response to uniform compression. It determines how much a compound reduces in volume when subjected to external pressure. High or low values of *B* indicate that the material is more or less resistant to fracture, respectively. The shear modulus measures a compound's response to shear deformation. High or low values of *G* show that the material is rigid or deforms quickly, respectively. The bulk and shear moduli are evaluated by applying the Voigt–Reuss model. The coefficients of the Voigt–Reuss model define the maximum and minimum values of the effective modulus, respectively. In a cubic system, the Voigt (*B*_V_) and Reuss (*B*_R_) bulk moduli, as well as Voigt (*G*_V_) and Reuss (*G*_R_) shear moduli, are calculated using expressions.^64^ The Hill theory^70^ states that *B* and *G* are obtained as the arithmetic means of Voigt–Reuss expressions. As AlMgCl_3_ has higher values of *B* and *G* than AlMgI_3_, AlMgCl_3_ has a greater fracture and deformation resistance than AlMgI_3_. Additionally, our computed results of *B* and *G* for AlMgX_3_ (X = Cl and I) are significantly higher than those for recently studied materials, such as CsCaX_3_ (X = F, Cl, Br)^69^ and A_3_PCl_3_ (X = Sr, Ba). A compound's stiffness is defined by the *Y*. It is associated with the *B* and *G* of the studied materials. The *Y* and *ν* ratios were determined by the equations described elsewhere.^64^ According to the calculated value of *Y*, AlMgCl_3_ is expected to be stiffer than AlMgI_3_.

**Fig. 3:**
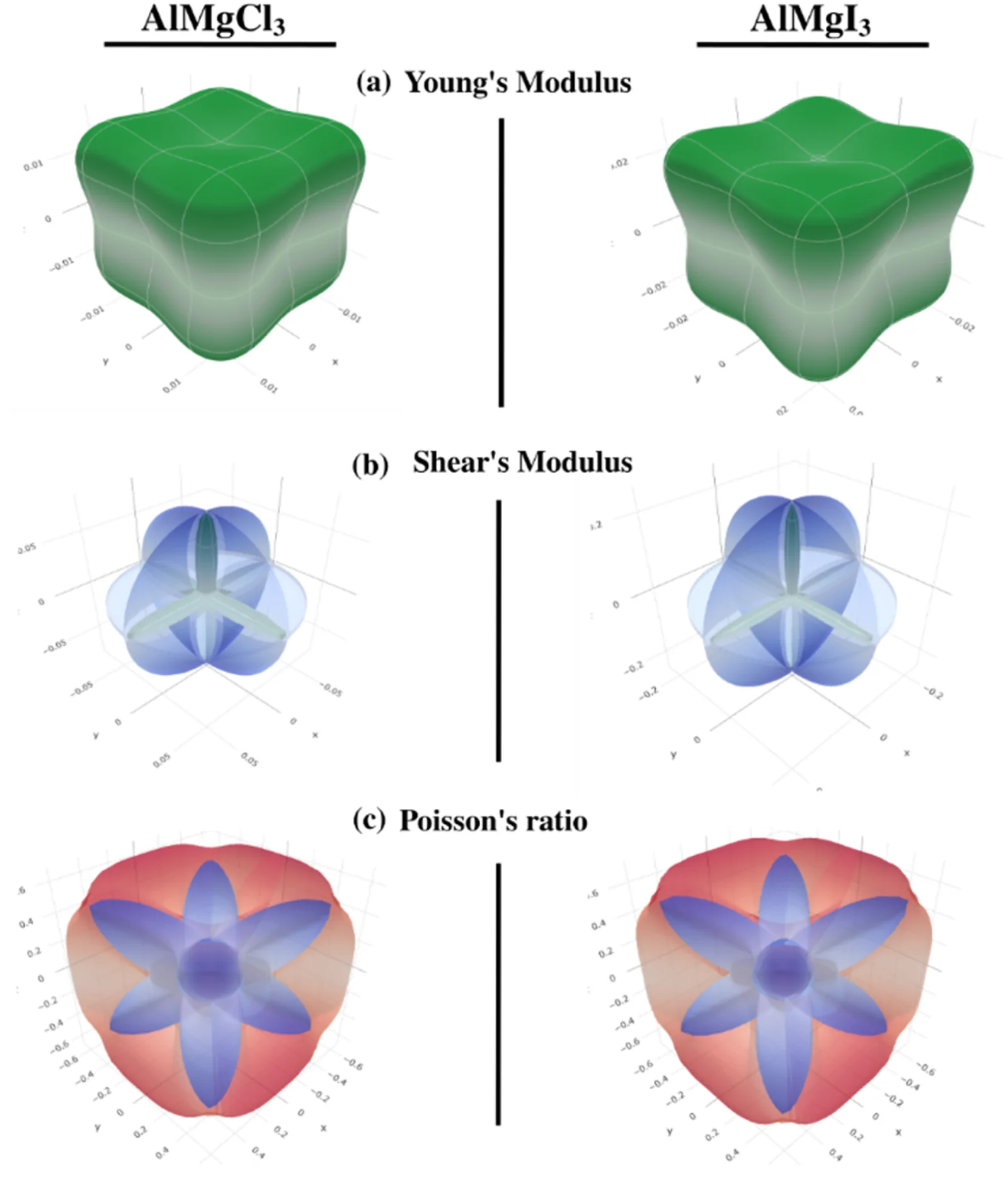
Representation of 3D anisotropic contours of (a) Young's modulus, (b) shear modulus, and (c) Poisson's ratio for AlMgCl_3_ and AlMgI_3_.

The *ν* is a material property that calculates the ratio of lateral strain to axial strain when a substance is compressed or stretched. The key value of *ν* used to differentiate whether a compound has a brittle or ductile nature is 0.26. A compound is said to be brittle or ductile if *ν* is lower or higher than 0.26, respectively. Therefore, both AlMgCl_3_ and AlMgI_3_ have a ductile nature. Another crucial parameter that can distinguish between ductility and brittleness is *B*/*G*. It has a critical value of 1.75 that defines a compound as brittle or ductile. Both studied compounds show a ductile nature according to Pugh's ratio. However, AlMgI_3_ is considered more ductile than AlMgCl_3_ due to higher values of *ν* (0.34) and *B*/*G* (2.97). It can be seen that when we change the halogen atom from Cl to iodine(i), the ductility of the studied material increases because the introduction of a heavy halogen atom, such as an iodine atom, weakens the bond strength, decreases the shear modulus, increases the *B*/*G* value, and increases the dislocation mobility, all of which contribute to higher ductility in AlMgI_3_. The variation in elastic behaviour with respect to the replacement of the halogen atom is graphically shown in Fig. 3.

Furthermore, compounds with a *ν* magnitude of 0.25 indicate the presence of an ionic character, whereas those with a *ν* magnitude of 0.1 mostly show covalent bonding.^71^ In the current study, both AlMgCl_3_ and AlMgI_3_ exhibit ionic bonding. Additionally, our findings on Pugh's and Poisson's ratios are consistent with recently reported data, such as GaXCl_3_ (X = Ca, Sr, Ba).^64^ In the current investigation, it was crucial to find the influence of *A* on these properties. A system's properties may be directionally dependent, which is quantified using *A*. The shear anisotropic factor identifies the extent of anisotropy in the bonding force of atoms across different crystallographic directions. The following shear anisotropic parameters, *A*_1_, *A*_2_, and *A*_3_, were computed across the (100), (010), and (001) crystallographic directions, respectively. In a cubic crystal system, these are all equivalent to the parameter *A* and can be calculated using the fundamental formula.^72^ An isotropic compound should have *A* = 1. If *A* > 1 or *A* < 1, the studied compound is said to be an anisotropic compound.^73^ The compounds AlMgCl_3_ and AlMgI_3_ have an anisotropic nature, which increases when the Cl atom is replaced with the iodine atom. So, AlMgI_3_ has more anisotropy than AlMgCl_3_. Fig. 3(a–c) reveals the orientation dependence of *G*, *Y* and *ν*, respectively, for AlMgCl_3_ and AlMgI_3_. Spherical 3D plots reflect isotropic behaviour, while non-spherical plots demonstrate anisotropy. The Zener anisotropy of AlMgCl_3_ and AlMgI_3_ appears in all possible orientations, as represented by asymmetric (non-spherical) shapes. The distortion of spherical shapes is more extreme for AlMgI_3_ than AlMgCl_3_, manifesting that the introduction of the iodine atom promotes the anisotropy of AlMgX_3_.

### 3.3 Electronic properties

**Fig. 4:**
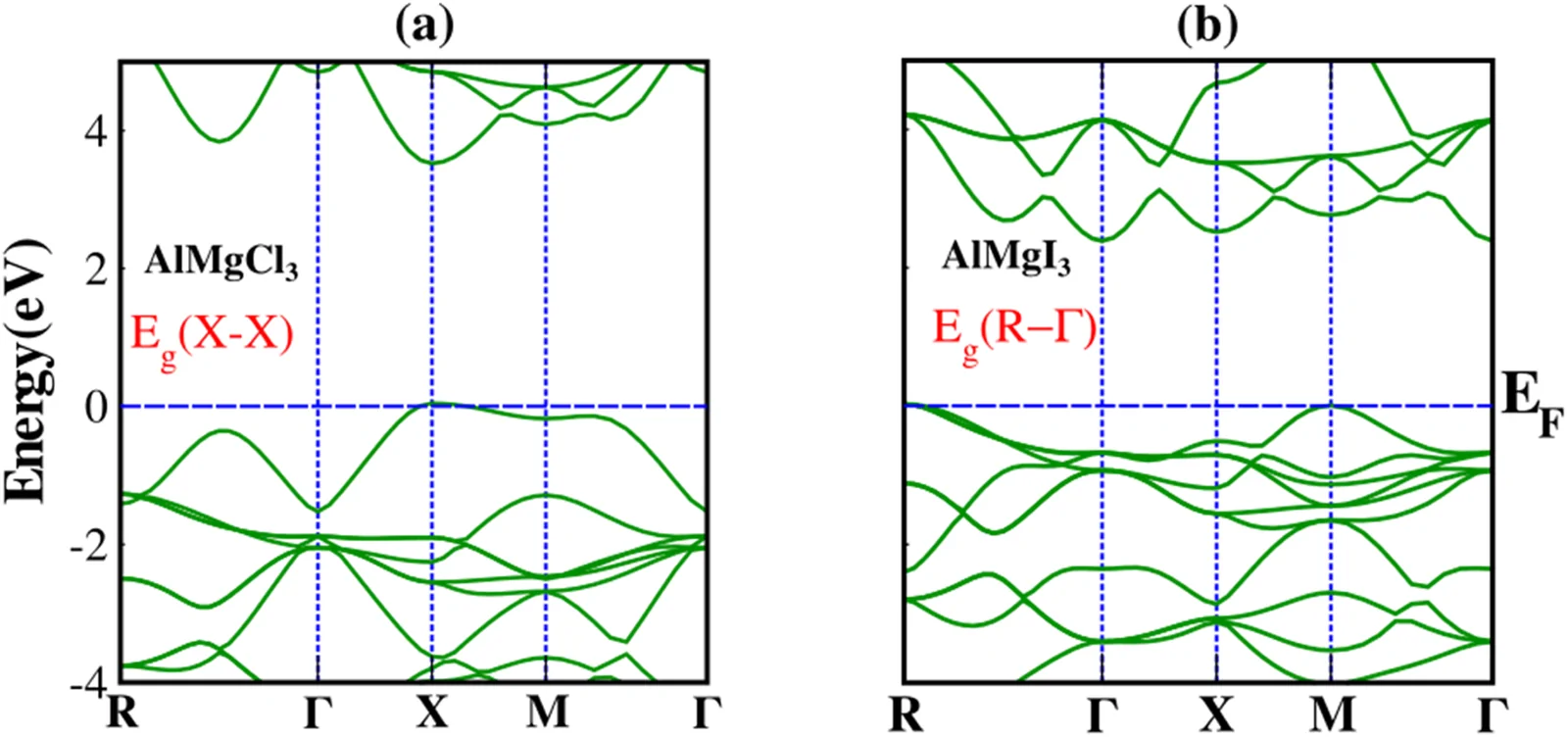
Computed electronic band structures of (a) AlMgCl_3_ and (b) AlMgI_3_.

A compound's electronic band structure (EBS) defines the allowed and forbidden energy levels for electron occupancy. This information is necessary to study the electronic characteristics of compounds, including magnetism, conductivity, carrier mobility rate, energy gaps and optical response.^74^ The EBS plays an important part in the growth and production of electronic components, including transistors, solar cells and LEDs. We calculated the EBSs of AlMgCl_3_ and AlMgI_3_ to know more about their electronic device efficiency. The precision of quantum simulations was based on the nature of the exchange and correlation potentials. The implementation of the generalized gradient approximation approach for EBS evaluation resulted in an underestimated value, a common problem of the generalized gradient approximation method. The underestimation of the EBS has been reported in LDA and (LDA) + U techniques.^75^ Researchers have suggested many methodologies, such as hybrid functionals^76,77^ as well as the Green and screened Coulomb interaction technique,^78^ to predict this form of energy gap analysis; however, these strategies also have certain limitations. The functional, which was defined by Heyd–Scuseria–Ernzerh ,^79^ can enhance the computed energy gap value to align more closely with experimental results; nevertheless, this is not universally applicable. In certain instances, the theoretically predicted value may be partly adjusted *via* the GGA with the Hubbard U correction.^80^ However, Nayak *et al.*^81,82^ recently showed that the PBE method reliably predicts strain-driven modifications in the EBS and energy gap, irrespective of the functional used. This signifies that the PBE/GGA functional is much better for studying the physical properties of materials. So, we used the PBE-GGA with the TB-mBJ functional to examine the influence of different halogen atoms on the energy gap value and the creation of the total/partial density of states (DOS). The EBS of AlMgX_3_ was calculated by considering high-symmetry *k*-points, such as *R*, *Γ*, *X*, *M* and *Γ*. The complete EBS repeats itself at these *k*-points. As presented in Fig. 4, the horizontal blue dotted lines denote the Fermi level (*E*_f_). From Fig. 4(a), it can be easily observed that the maximum and minimum locations of the valence (VB) and conduction bands (CB) of AlMgCl_3_ are situated at the same symmetry point, which is designated as X. This situation of the EBS of AlMgCl_3_ clearly exhibits the direct nature of the studied compound with an energy bandgap (*E*_g_) value of 3.60 eV. A crystalline solid material having a direct nature of the EBS is commonly favored for photothermal, optoelectronic and photovoltaic applications. However, after we replace the X atom with an iodine atom in AlMgX_3_, the nature of the EBS is quite different from that observed in AlMgCl_3_. The introduction of the iodine atom (AlMgI_3_) reduces the *E*_g_ value as well as changes the nature of the EBS from direct (X–X) to indirect (*R*–*Γ*), with an *E*_g_ value of 2.44 eV. The decrease in *E*_g_ facilitates the efficient movement of electrons from the VB to the CB. Consequently, the polarization and optical absorption may significantly enhance the efficacy in optoelectronic applications. The behaviour of the EBS changes upon replacing the halogen atom chlorine to iodine due to different physical factors such as the atomic size and bond strength, the reduction in bond ionicity, and the spin–orbit coupling effect. Firstly, we will explain how the atomic size and bond strength can change the nature of the EBS. As we know, iodine is a larger anion than Cl, which has a lower electronegativity. This factor reduces the bonding strength between Al–I and Mg–I, which leads to smaller orbital overlap. This weak bonding among the atoms reduces the energy gap between the VB and the CB. Moreover, the incorporation of the heavy iodine atom affects orbital interactions and disturbs the crystal symmetry; as a result, the electronic states of AlMgI_3_ are modified, which favours the indirect band gap by breaking the direct transitions. Secondly, a reduction in bond ionicity also affects the electronic band structure. As we discussed earlier, the Cl atom has a higher electronegativity value (3.16) than the I atom (2.66). So, when we introduce the iodine atom, the bond becomes more ionic and less covalent. The ionic nature of bonding usually reduces the *E*_g_ value because the CB shifts downward, which lowers the energy gap. Thirdly, the EBS of a compound also depends on the spin–orbit coupling effect. The iodine atom exhibits a stronger spin–orbit coupling effect than Cl owing to its higher atomic number. The VB and CB are split by this spin–orbit interaction, which can also modify the EBS from direct to indirect. Additionally, our calculated values of *E*_g_ are comparable with those of recently studied lead-free perovskite materials such as GaXCl_3_ (2.92–3.45 eV)^64^ and AlMgBr_3_ (3.95 eV).^39^ Additionally, we performed the mBJ + SOC calculation because a heavy element like iodine can significantly affect the electronic band gap. We observed a slight increase of 0.27% and 2.82% in the band gaps of AlMgCl_3_ and AlMgI_3_, respectively.

**Fig. 5:**
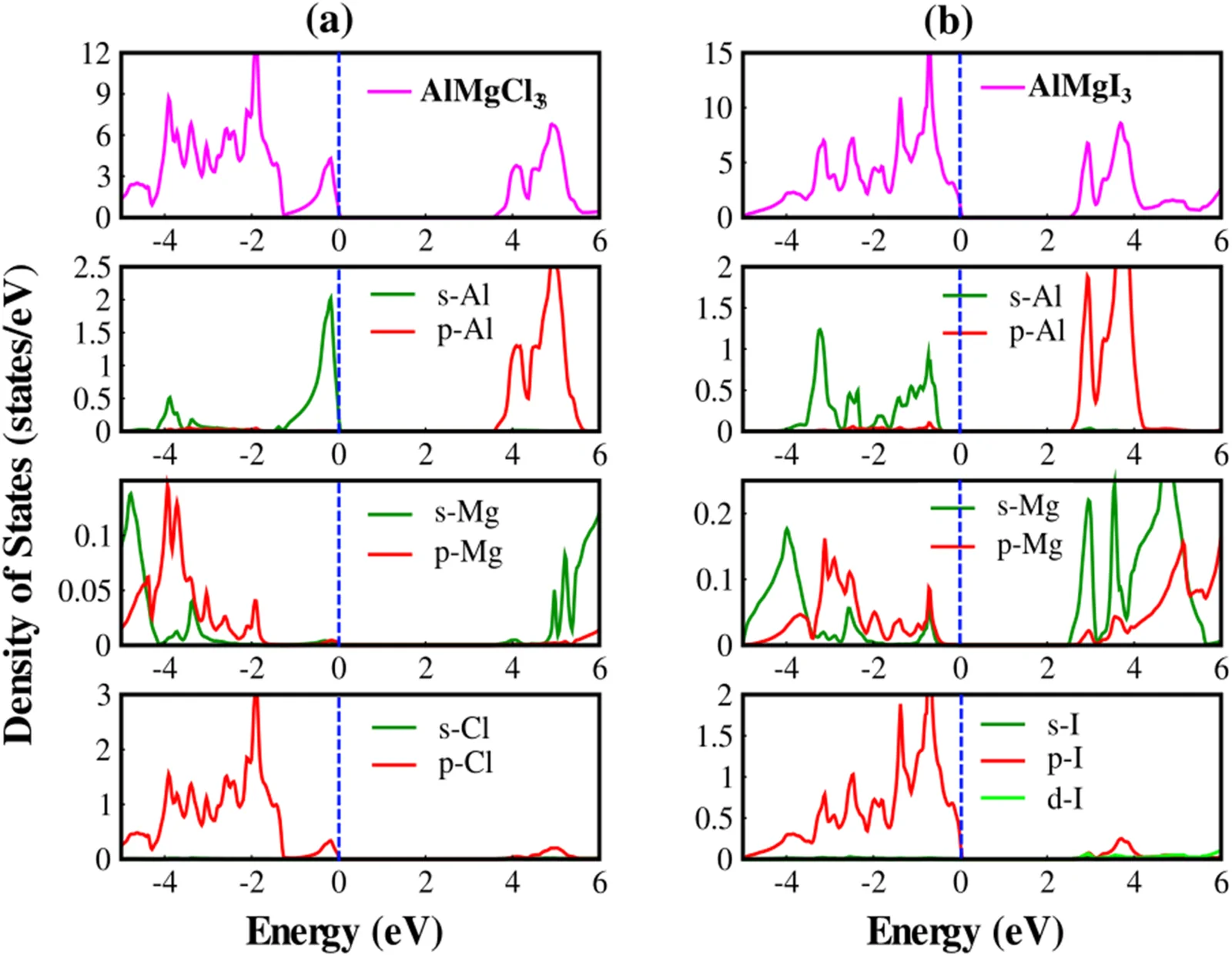
Computed density of states of (a) AlMgCl_3_ and (b) AlMgI_3_.

To further examine the electronic properties, Fig. 5 exhibits the total/partial DOS of AlMgX_3_, which we determined based on the EBS as well as the contribution of electrons from various states. As shown in Fig. 5, the absence of electronic states near the *E*_f_ indicates a bandgap as well as the intrinsic semiconductor character of AlMgX_3_. The lack of TDOS from the Fermi level to 3.60 eV and 2.44 eV actually defines the values of *E*_g_ for AlMgCl_3_ and AlMgI_3_, respectively.

Replacing the halogen atom has a significant effect on the TDOS in the CB, and all the significant peaks gradually shift towards the *E*_f_ when we replace the Cl atom with the iodine atom. The peak shifting causes the band gap to decrease, as seen in the EBS (Fig. 4) at the *R*- and *Γ*-points. Fig. 5(a and b) present the total/projected DOS of AlMgCl_3_ and AlMgCl_3_ in the energy range from −5 eV to 6 eV, respectively. The maximum value of the TDOS of AlMgCl_3_ is calculated to be 12 states per eV at −2 eV. However, after the incorporation of the iodine atom as the X atom, the TDOS of AlMgI_3_ rises significantly and reaches the maximum value (15 states per eV) at −1 eV. The projected DOS is essential for determining the atomic contributions of a compound in constructing its band diagram. Fig. 5(a and b) clearly demonstrate that the VBs of AlMgCl_3_ and AlMgI_3_ compounds are near the *E*_f_, mostly due to Al-s and Cl-/I-p orbitals, with a little contribution from Mg-p orbitals. In contrast, the CB mainly originates from the Al-p and Mg-s orbitals, with a minor contribution from Mg-p and Cl-/I-p states. It can be seen that the Al-p and I-p orbitals mostly contribute to the reduction of *E*_g_ in the AlMgI_3_ compound. The hybridization between Al-p and I-p is influenced by replacing the chlorine atom with iodine, which elevates the CB towards the *E*_f_ and lowers the band gap. A similar trend of band gap reduction has also been observed in the recently reported perovskite material CsCaX_3_ (X = F, Cl, Br).^69^

### 3.4 Optical properties

Optical characteristics are important factors that may be examined by the distinctive behaviour of photon energy, determining the suitability of a compound for applications, including optoelectronic devices and solar cells. Pb-free metal halide ABX_3_ compounds have gained significant attention from researchers owing to their interesting optical properties. They exhibit remarkable performance in optical and solar cell devices. The most important optical parameters, like dielectric function (*ε*(*ω*)), refractive index (*n*(*ω*)), extinction coefficient (*k*(*ω*)), reflectivity (*R*(*ω*)) and optical conductivity (*σ*(*ω*)), were computed for AlMgCl_3_ and AlMgI_3_ compounds to examine their efficiency in PV and optoelectronic devices. This study investigated and calculated the optical response of AlMgX_3_ (X = Cl and I) from 0 eV to 12 eV. The angular frequency-based dielectric function, closely associated with the EBS of the AlMgX_3_ compounds (X = Cl and I), may be computed using the following relation: *ε*(*ω*) = *ε*_1_(*ω*) + *iε*_2_(*ω*). Here, *ε*_1_(*ω*) defines the real component, and *ε*_2_(*ω*) signifies the imaginary component of the dielectric function. The Kramers–Kronig^56^ relation was used to find *ε*_1_(*ω*).1
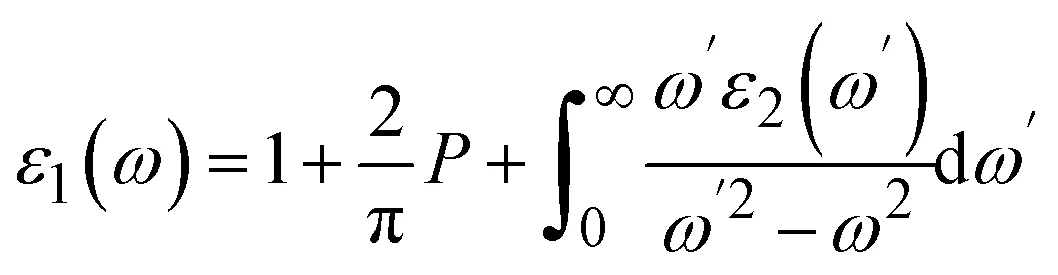


*ε*_2_(*ω*) is related to the momentum tensor during the transition of electrons from filled to unfilled states and may be obtained using the following expression:^83,84^2
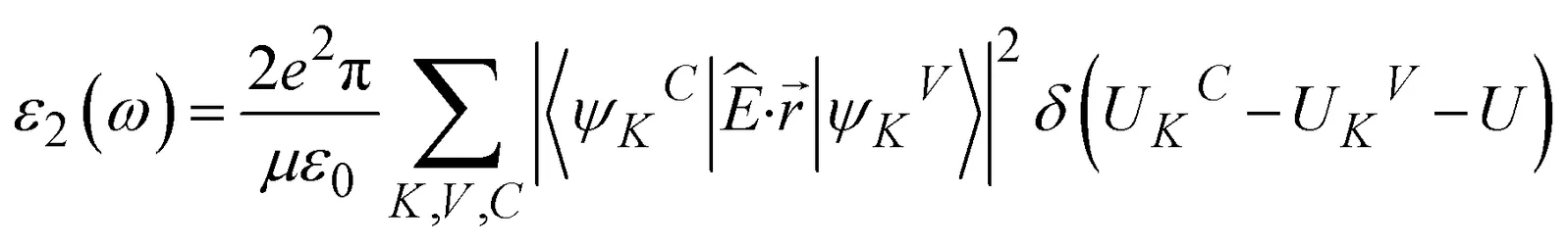
Here, *ω* is the light angular frequency. *ψ*_*K*_^*V*^ denotes the wavefunction of the VB, and *ψ*_*K*_C represents the wavefunction of the CB at *k*. *e* defines the electronic charge, *µ* is the unit cell volume, and *E* is the polarization of the electric field. *U*_*K*_^*C*^ and *U*_*K*_V represent the electronic energies in the CB and VB at the *k*-point, respectively.

**Fig. 6:**
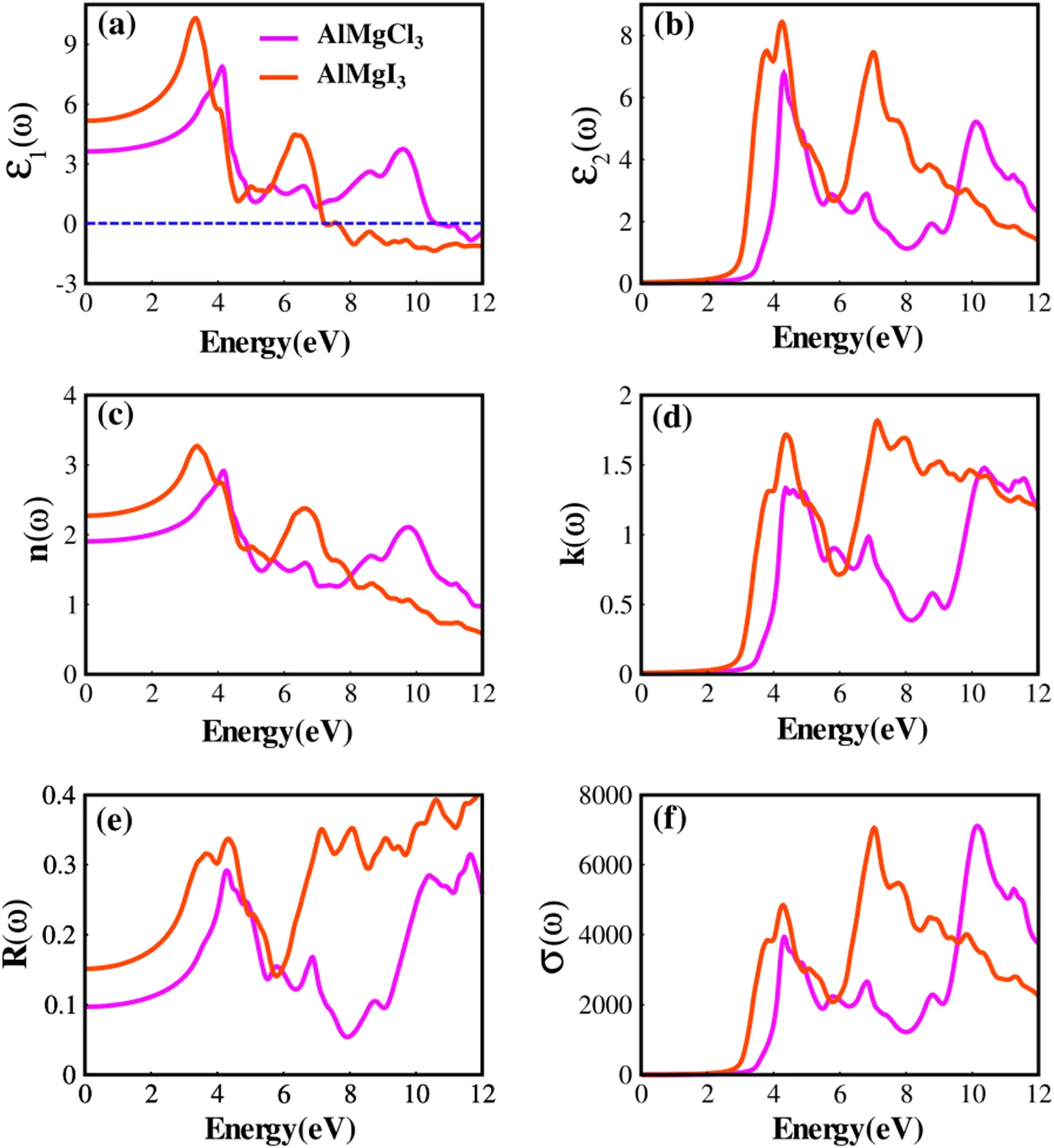
Calculated optical properties: (a) real part (*ε*_1_(*ω*)), (b) imaginary part (*ε*_2_(*ω*)), (c) refractive index (*n*(*ω*)), (d) extinction coefficient (*k*(*ω*)), (e) reflectivity (*R*(*ω*)), and (f) optical conductivity (*σ*(*ω*)) of AlMgX_3_.

The computed values of *ε*_1_(*ω*) of AlMgX_3_ (X = Cl and I) are depicted in Fig. 6(a) for energies ranging from 0 to 12 eV. *ε*_1_(*ω*) describes the dispersion and polarization phenomena of the studied material. *ε*_1_(*ω*) is often referred to as the static dielectric function at zero angular frequency, *ε*_1_(0). This *ε*_1_(0) reveals an inverse relation with the EBS using Penn's model. As the value of *E*_g_ lowers, *ε*_1_(0) increases significantly, which supports Penn's model. From Fig. 6(a), it can be seen that the static values of *ε*_1_(*ω*) for AlMgCl_3_ and AlMgI_3_ are 3.55 and 5.14, respectively. The value of *ε*_1_(0) for AlMgCl_3_ is comparable to that of recently reported halide-based perovskites such as GaXCl_3_ (3.2–3.8)^64^ and AlMgBr_3_ (3.27),^39^ but AlMgI_3_ has a much higher value than these reported data. AlMgI_3_ with the maximum value of *ε*_1_(0) exhibits a minimum carrier recombination rate. So, AlMgI_3_ has potential for photovoltaic device applications. Moreover, the real component (*ε*_1_(*ω*)) has maximum polarization values of 7.8 (4.1 eV) and 10.2 (3.32 eV) for AlMgCl_3_ and AlMgI_3_, respectively. However, as the photonic energy rises to a higher value, *ε*_1_(*ω*) drops to a minimum value and becomes negative in the higher UV energy regions of 10.9–12 eV and 7.7–12 eV for AlMgCl_3_ and AlMgI_3_, respectively. The negative magnitudes of the real component demonstrate the materials' reflective nature to the incoming photons, confirming their metallic characteristics, which makes AlMgCl_3_ and AlMgI_3_ suitable for UV-shielding applications.^85^ To provide a more comprehensive benchmark, the optical properties of AlMgX_3_ (X = Cl and I) were compared with those of well-known halide perovskite systems reported in the literature. The calculated static dielectric constants, *ε*_1_(0), for AlMgCl_3_ (3.55) and AlMgI_3_ (5.14) lie within the typical range for lead-free halide perovskites and double perovskites such as Cs_2_AgBiBr_6_ (∼4.0–6.0) and Tl-based halide perovskites (∼3.2–5.0).^86^ The observed increase in the dielectric constant with decreasing band gap is consistent with general trends reported in halide perovskite systems and is in agreement with the Penn model.^87^ The negative values of *ε*_1_(*ω*) in the higher ultraviolet region indicate strong reflection and electromagnetic attenuation, similar to the behavior reported for other halide perovskites used for UV-shielding and optical coating applications. Overall, the presented results show that AlMgX_3_ compounds exhibit optical characteristics comparable to those of established halide perovskite materials, supporting their potential relevance for UV-visible optoelectronic applications.

*ε*_2_(*ω*) was used to define the absorption and energy gain within AlMgCl_3_ and AlMgI_3_ compounds. Fig. 6(b) exhibits the response of *ε*_2_(*ω*) of AlMgCl_3_ and AlMgI_3_ compounds in the energy range of 0–12 eV. very low photonic energy, the studied halide-based perovskites show very low absorption, which may be associated with the calculated EBS. In the case of AlMgCl_3_, the absorption starts from 3.4 eV. However, as the energy of the incoming photon increases, the absorption within the AlMgCl_3_ material also increases and approaches the maximum value of 6.70 at 4.34 eV. After this energy value, *ε*_2_(*ω*) decreases to the minimum value because the number of available electronic states decreases to allow inter-band transitions. Furthermore, different absorption peaks in *ε*_2_(*ω*) at different angular frequencies reveal that electronic excited states do not just correlate with rising energy; rather, they depend on the computed EBS, where vacant and filled electronic states are defined by a certain energy gap. However, after we replace the Cl atom with the iodine atom (AlMgI_3_), the absorption peaks rise significantly and also shift toward the lower-energy region, which is known as the visible region. The absorption spectra shift towards the lower-energy range, resulting in the reduction of *E*_g_. In AlMgI_3_, absorption starts from 2.98 eV, which rises to the maximum value (8.41) in the energy region of 2.99–4.3 eV. This suggests that AlMgI_3_ absorbs light well in both visible and lower UV regions. According to the solar EM spectrum, photons with an energy less than 3.4 eV account for 98% of the solar energy reaching Earth.^88^ The significant values of *ε*_2_(*ω*) for AlMgCl_3_ and AlMgI_3_ in lower-energy regions make them ideal for solar cells, photodetectors and integrated circuits.^89^

The expression *N*(*ω*) = *n*(*ω*) + *ik*(*ω*) was used to define the complex refractive index. Here, the real component, *n*(*ω*), is called the refractive index, and the imaginary part, *k*(*ω*), is defined as the extinction coefficient. Both *n*(*ω*) and *k*(*ω*) were evaluated using the following equations:^87^3
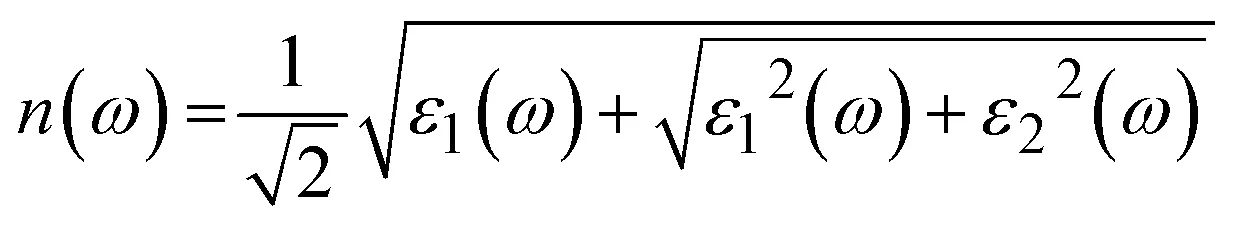
4
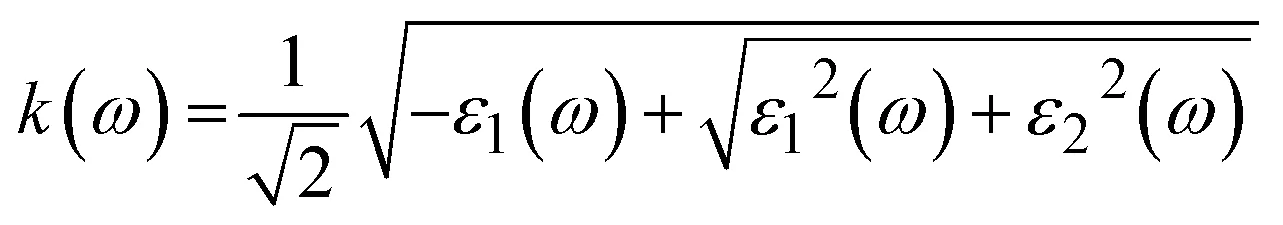


The optical response of AlMgCl_3_ and AlMgI_3_ in terms of *n*(*ω*) and *k*(*ω*) is plotted in Fig. 6(c and d), respectively. *n*(*ω*) measures the degree of light bending or refraction when it enters or exits a material. According to eqn (3), both *ε*_1_(*ω*) and *n*(*ω*) have almost the same nature of optical spectra, as depicted in Fig. 6(a and c), respectively. The computed values of the static refractive index, *n*(0), for AlMgCl_3_ and AlMgI_3_ are 1.90 and 2.27, respectively, which follow the relation *n*(0) = *ε*_1_^2^(0). Materials with a high refractive index may efficiently regulate the propagation of light in optical waveguides and improve the functionality of optical systems. At 0 eV, the value of *n*(*ω*) exceeds unity because the photon velocity decreases after entering a substance. As an increasing number of photons interact with the electrons while traversing the material, the photons experience a higher deceleration, resulting in an elevated refractive index and an enhanced electron density. The maximum value of *n*(*ω*) can be seen in the UV and visible regions for AlMgCl_3_ and AlMgI_3_, respectively; however, it decreases to the minimum value in the higher UV region. *k*(*ω*) gives information about a material's potential to attenuate incident photons.^90^ The extinction coefficient is related to the imaginary component of the dielectric function. It indicates that absorption has the maximum value where refractivity has the minimum value, which makes AlMgCl_3_ and AlMgI_3_ opaque. The *n*(*ω*) values at *n*(0) of 1.90 (AlMgCl_3_) and 2.27 (AlMgI_3_) are also comparable to those of other halide perovskites such as MAPbI_3_ (∼2.3–2.6)^91^ and CsPbBr_3_ (∼2.2–2.4),^92^ indicating similar light–matter interaction strengths. Furthermore, the optical absorption edge of AlMgI_3_ is red-shifted toward the visible region, consistent with trends observed in iodide-based perovskites such as MAPbI_3_ and CsSnI_3_, which exhibit strong visible-light absorption due to their narrower band gaps.

Reflectivity (*R*(*ω*)) is another important optical feature that provides insights into the surface roughness of a compound to incoming photons. The calculated values of *R*(*ω*) at zero angular frequency (static value) are 9.63% and 15% for AlMgCl_3_ and AlMgI_3_, respectively. At a low frequency, *R*(*ω*) is low because most of the light is transmitted due to the absence of electronic transitions. However, as the photonic energy rises, the movement of electrons from the VB to the CB also increases; as a result, the value of *ε*_1_(*ω*) also increases, leading to a higher reflectivity. Above 4.2 eV, the reflectivity decreases sharply because the imaginary part of the dielectric constant rises, which means light is absorbed instead of being reflected by the materials. At the critical value (∼12 eV) of energy, the maximum *R*(*ω*) is calculated to be 26.1% and 40% for AlMgCl_3_ and AlMgI_3_, respectively. The relatively minimum value of *R*(*ω*) in the low-energy range suggests the potential of AlMgCl_3_ and AlMgI_3_ for solar cell manufacturing. Furthermore, these compounds can be used as coating materials to reduce solar heating due to their highest reflectivity in the high-energy spectrum.

*σ*(*ω*) results from the capture of incoming photons, which facilitate electronic transitions to higher energy states, thereby establishing a direct relationship with absorption. The optical conductivity demonstrates a trend aligned with the parameters, indicating the absorption of the light, as depicted in Fig. 6(b). Two prominent peaks of *σ*(*ω*) at different energies can be seen in Fig. 6(f) for AlMgCl_3_ and AlMgI_3_ compounds. The calculated peak values are 3912 (4.3 eV), 7105 (10.16 eV) and 4853 (4.3 eV), 7027 (7.07 eV) for AlMgCl_3_ and AlMgI_3_, respectively. It is clear that the *σ*(*ω*) of AlMgX_3_ rises significantly after we replace the Cl atom with the iodine atom, which is in good agreement with the recent investigation of bandgap narrowing due to a change in the halogen atom.

### 3.5 Thermoelectric properties

**Fig. 7:**
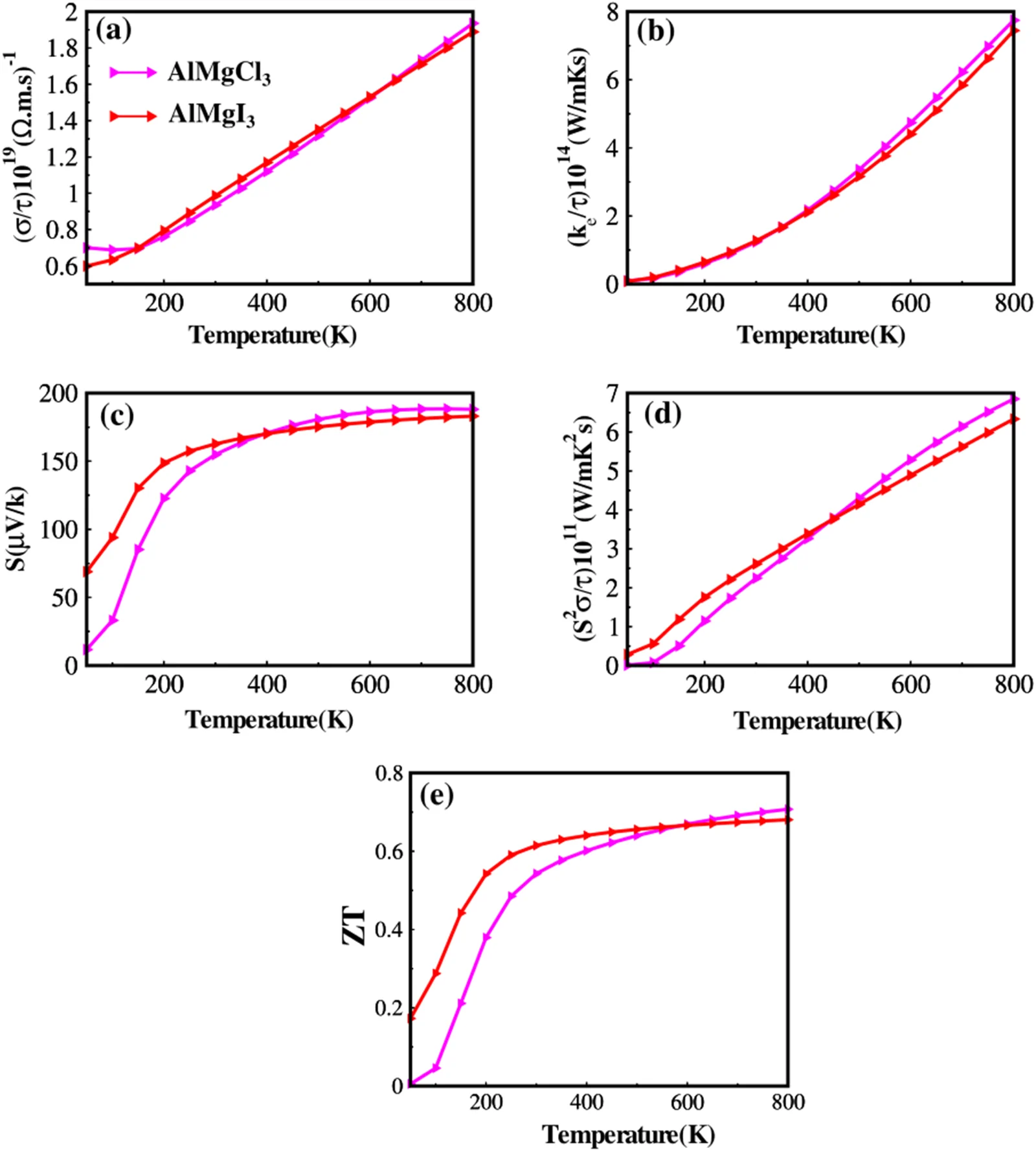
Calculated thermoelectric characteristics *vs.* temperature in terms of (a) electrical and (b) thermal conductivities, (c) Seebeck coefficients, (d) power factors and (e) figures of merit of AlMgX_3_.

Scientists are focusing on developing materials that can extract energy from sustainable sources and retain it for long periods. Thermoelectric (TE) compounds have garnered significant attention in this regard. Highly efficient TE materials are essential to harness the heat supplied by any system. The performance of any TE material is based on the nature of the EBS and the type of charge carriers that facilitate electrical conduction. Herein, we investigated the TE response of AlMgX_3_ (X = Cl and I) by evaluating parameters such as electrical conductivity (*σ*/*τ*), electronic thermal conductivity (*κ*_e_/*τ*), Seebeck coefficient (*S*), power factor (PF), figure of merit (*ZT*) and lattice thermal conductivity (*κl*). Here, *τ* represents the relaxation time. The temperature-dependant TE properties of AlMgCl_3_ and AlMgI_3_ are plotted in Fig. 7.

Fig. 7(a) presents the electrical conductivity (*σ*/*τ*) behaviour of AlMgCl_3_ and AlMgI_3_ compounds in the temperature range of 50–800 K. *σ*/*τ* defines the potential of a material to transport electric charges when a potential difference is applied. It plays an important role in determining the ability of a compound to convert heat energy into electric current. The *σ*/*τ* of AlMgCl_3_ and AlMgI_3_ rises linearly with temperature, which reveals a negative temperature coefficient of resistance because the value of resistance decreases as the temperature rises. It can be noticed that at the lowest temperature (50 K), AlMgCl_3_ and AlMgI_3_ compounds have *σ*/*τ* values of 0.70 × 10^19^ (Ω ms)^−1^ and 0.59 × 10^19^ (Ω ms)^−1^, respectively. However, as the temperature increases from 50 K to room temperature (300 K), the value of *σ*/*τ* also rises to 0.93 × 10^19^ (Ω ms)^−1^ and 0.98 × 10^19^ (Ω ms)^−1^ for AlMgCl_3_ and AlMgI_3_, respectively. At 800 K, the electrical conductivity of AlMgCl_3_ and AlMgI_3_ compounds reaches the maximum value, which is calculated to be 1.93 × 10^19^ (Ω ms)^−1^ and 1.88 × 10^19^ (Ω ms)^−1^, respectively. In fact, in semiconducting materials, *σ*/*τ* depends on the thermal energy required to excite charge carriers across the electronic bandgap. At 50 K, AlMgCl_3_ and AlMgI_3_ compounds have insufficient thermal energy to transit the electrons from the VB to the CB, resulting in lower electrical conductivity. However, as the temperature increases to the maximum value, electrons gain sufficient energy to make the transition from the VB to the CB; as a result, the availability of free charge carriers increases. This intrinsic charge carrier concentration obeys the following relation: *n*_*i*_ ∝ *e*^−*E*_g_/2*k*_B_*T*^.^93^ Here, *n*_*i*_ is the intrinsic concentration of charge carriers, *E*_g_ signifies the energy bandgap value, *k*_B_ is Boltzmann's constant and *T* is the absolute temperature. This is why the value of electrical conductivity is observed to be the minimum at lower temperatures and the maximum at higher temperatures for AlMgCl_3_ and AlMgI_3_. Furthermore, it has been noticed that the value of *σ*/*τ* for AlMgI_3_ is greater than that for AlMgCl_3_ at room temperature because AlMgCl_3_ has a low carrier concentration due to a wide electronic bandgap (according to the above-mentioned relation) compared to AlMgI_3_. However, at a temperature of 800 K, the value of *σ*/*τ* for AlMgI_3_ is lower than that for AlMgCl_3_ due to the indirect nature of the energy bandgap of AlMgI_3_. Because at higher temperatures, the interactions of electrons with phonons increase, the transition of electrons across the bandgap decreases. This effect may reduce the electrical conductivity of AlMgI_3_ at higher temperatures.

The thermal conductivity (*κ*_e_/*τ*) is defined by the potential of a material to conduct heat. Both electrons and phonons play a crucial role in describing the phenomenon of heat transmission in a material. The computed values of *κ*_e_/*τ* for AlMgCl_3_ and AlMgI_3_ with respect to temperature variations are shown in Fig. 7(b). *σ*/*τ* and *κ*_*e*_/*τ* exhibit almost similar trend because only the electronic part is considered. At a low temperature (50 K), *κ*_e_/*τ* is computed to be 0.09 × 10^14^ (W mKs^−1^) and 0.08 × 10^14^ (W mKs^−1^) for AlMgCl_3_ and AlMgI_3_, respectively. As the temperature rises to 300 K, the value of *κ*_e_/*τ* becomes 1.24 × 10^14^ (W mKs^−1^) and 1.27 × 10^14^ (W mKs^−1^), which further increases to 7.74 × 10^14^ (W mKs^−1^) and 7.44 × 10^14^ (W mKs^−1^) at a temperature of 800 K for AlMgCl_3_ and AlMgI_3_, respectively. From Fig. 7(b), it can be seen that both compounds, AlMgCl_3_ and AlMgI_3_, exhibit almost similar electronic thermal conductivity responses up to 400 K because AlMgCl_3_ and AlMgI_3_ have low-density charge carriers at low temperatures. So, their electronic thermal conductivity behaviour is identical in this temperature range. Above 400 K, the trend of *κ*_e_/*τ* for AlMgCl_3_ deviates from that of AlMgI_3_. At high temperatures, in AlMgI_3_, as the carrier concentration rises, the electron and phonon interactions become more significant, which may enhance the electrical resistivity and affect *κ*_e_/*τ*. Generally, compounds with low *κ*_e_/*τ* are considered more suitable as cooling systems or heat sinks. This is crucial for electronic devices, like CPUs, GPUs and power control systems, to avoid overheating and achieve maximum efficiency. By contrast, compounds having high *κ*_e_/*τ* serve to maintain a constant temperature over the surfaces, preventing thermal gradients and localized hotspots that may degrade the performance of electronic devices and their reliability. Efficient heat management using compounds with maximum *κ*_e_/*τ* can increase energy efficiency by eliminating the need for unnecessary cooling devices. Effective heat management is a challenge for microelectronic systems, where space is limited. Compounds having high *κ*_e_/*τ* facilitate the design of heat spreaders, mini-heat sinks and thermally conductive materials that capably release heat energy from compact components. Thus, based on this fact, our studied materials may show potential for applications involving heat control.

The Seebeck coefficient (*S*) and power factor (PF) were evaluated in order to check the capability of TE materials. The Seebeck coefficient (*S*) measures the value of the induced TE voltage (Δ*V*) with respect to a temperature gradient (Δ*T*) through a material. The calculated values of *S* have been plotted *versus* temperature in Fig. 7(c). At 50 K, both compounds show the minimum values of the Seebeck coefficient because at very low temperatures, few charge carriers are thermally excited. As the temperature rises to room temperature, charge carriers get more energy to jump from the VB to the CB; as a result, the thermoelectric voltage increases, which enhances the Seebeck coefficient value. At room temperature, the highest magnitudes of *S* are recorded as 154.92 µV K^−1^and 162.72 µV K^−1^ for AlMgCl_3_ and AlMgI_3_, respectively. So, compounds with a greater *S* value are considered best for thermoelectric generators and coolers.^94^ After 300 K, the value of the Seebeck coefficient becomes almost constant for both compounds because at this stage, the charge carrier concentration becomes saturated. This means that as we further increase the temperature, no TE voltage is induced within the studied materials. Additionally, the trend of *S* for AlMgCl_3_ is observed to be totally different from that for AlMgI_3_ at different temperatures because AlMgCl_3_ has a different electronic band structure compared to AlMgI_3_, as explained in the electrical conductivity discussion above. However, the positive magnitudes of *S* suggest the p-type nature of the semiconducting materials (AlMgCl_3_ and AlMgI_3_).^95^

The power factor (PF = *σS*^2^) was calculated by taking the product of electrical conductivity and the square of the Seebeck coefficient (*S*). Fig. 7(d) exhibits the TE response of the studied materials in terms of the power factor in the temperature range of 50–800 K. The PF has an almost-linear relation with temperature, and the values of the PF for AlMgCl_3_ and AlMgI_3_ are obtained as 2.24 × 10^11^ W m^−1^ K^−2^ s^−1^ and 2.61 × 10^11^ W m^−1^ K^−2^ s^−1^, respectively, at 300 K. The value of the PF increases as the temperature rises, and the highest magnitude of the PF is calculated to be 6.84× 10^11^ W m^−1^ K^−2^ s^−1^ and 6.33 × 10^11^ W m^−1^ K^−2^ s^−1^ for AlMgCl_3_ and AlMgI_3_, respectively, at 800 K. Thus, it is clear that the PF rises with temperature, suggesting the possible application of the investigated lead-free metal halide perovskites even at high temperatures under stable phase conditions.

The figure of merit, *ZT*, is another important parameter of TE characteristics used to determine the potential of TE materials. To compute the *ZT* value of AlMgX_3_ (X = Cl and I), we consider the following mathematical relation: *ZT* = *S*^2^*σT*/*K*. The trends of the figure of merit for AlMgX_3_ (X = Cl and I) are plotted in Fig. 7(e). *ZT* is associated with the electronic band structure of the studied materials. It can be observed from Fig. 7(e) that after replacing the chlorine atom with I, the value of *ZT* increases significantly at 300 K because the introduction of the iodine atom reduces the energy band gap. The values of *ZT* are observed to be 0.54 and 0.61 for AlMgCl_3_ and AlMgI_3_, respectively, at 300 K. However, when we raise the temperature to 800 K, the value of *ZT* for AlMgI_3_ (0.68) decreases compared to that of AlMgCl_3_ (0.70) due to the indirect bandgap nature of AlMgI_3_, which involves phonons at high temperatures. However, these high values of *ZT* suggest that our studied materials have potential for the production of electrical energy by harnessing thermal energy. Furthermore, our computed *ZT* values at room temperature and 800 K are comparable to those of recently reported p-type materials such as ZnZrO_3_,^96^ Rb_2_AgPX_6_ (0.76)^65^ and X_2_AuYZ_6_ (0.51–0.75).^97^

**Fig. 8:**
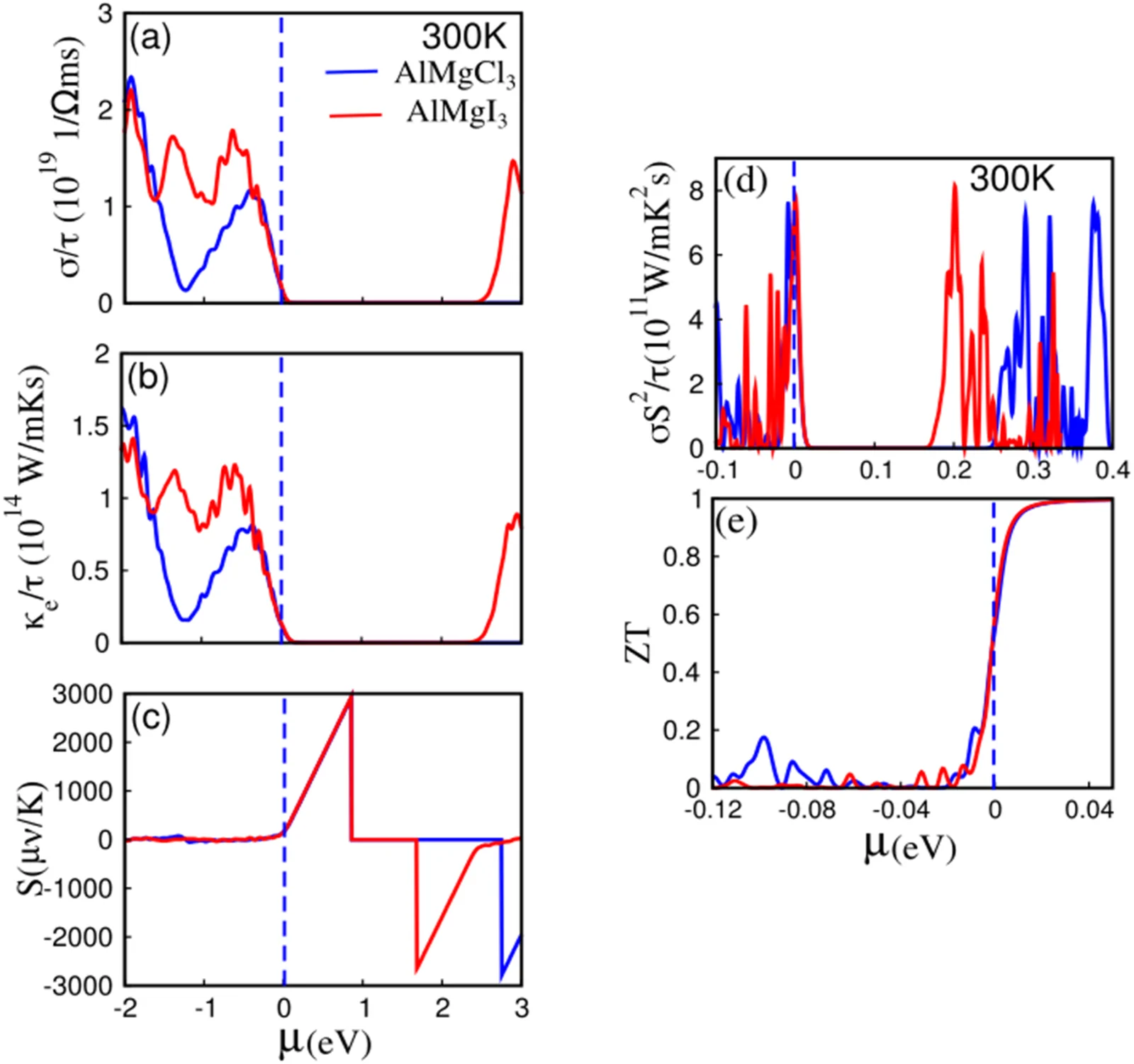
Calculated thermoelectric characteristics *vs.* chemical potential in terms of (a) electrical and (b) thermal conductivities, (c) Seebeck coefficients, (d) power factors and (e) figures of merit of AlMgCl_3_ and AlMgI_3_.

Fig. 8 presents the thermoelectric properties of AlMgX_3_ (X = Cl and I) against the chemical potential in the range from −2 eV to 3 eV. The chemical potential defines the change in the energy of a system when particles enter or leave, and it measures the material's reacting, phase-changing and diffusing abilities due to a concentration difference between two regions.^98^ For this purpose, the BoltzTrap source code was utilized. A good thermoelectric compound is one that has a lower ratio of thermal and electrical conductivities.^99^ Positive and negative values of n-type and p-type sites were utilized to represent the energy required for electrons and holes to be shifted to create the effective potential. Here, electrical conductivity (*σ*/*τ*) measures the charge transport due to both electrons and holes. Fig. 8(a) shows the variation in electrical conductivity of AlMgX_3_ (X = Cl and I) with respect to the chemical potential (*µ*). At room temperature, the highest values noted for the p-type region are found to be 2.2 × 10^19^ (1 Ω ms)^−1^ and 2.3 × 10^19^ (1 Ω ms)^−1^ for AlMgCl_3_ and AlMgI_3_, respectively, at −1.9 eV, showing the maximum availability of holes for conductivity in these structures, which may be due to larger-ionic-radius Cl and I atoms. Additionally, carrier mobility in the n-type region starts at 2.4 eV for AlMgCl_3_ and has the maximum contribution of 1.6 × 10^19^ (1 Ω ms)^−1^ at 2.8 eV, which is due to the maximum density of states near the conduction band minimum and the lower effective mass of electrons, whereas AlMgI_3_ has no contribution in the n-type region. Fig. 8(b) presents the variation of thermal conductivity (*κ*_e_) *vs.* chemical potential (*µ* (eV)) at room temperature (300 K). The highest values of this parameter are found to be 1.35 × 10^14^ W m^−1^ K^−1^ s^−1^ and 1.6 × 10^14^ W m^−1^ K^−1^ s^−1^ for AlMgCl_3_ and AlMgI_3_, respectively, at −1.8 eV and −2 eV, respectively, which may be due to the existence of the valence band maximum near the Fermi level and the dominance of the hole carrier concentration. In the n-type region, thermal conductivity starts at around 2.4 eV and increases sharply till it reaches 1 × 10^14^ W m^−1^ K^−1^ s^−1^ at around 2.8 eV, which may be due to the existence of the CBM near the Fermi level and the higher electron concentration for transport. Fig. 8(c) demonstrates the variation of the Seebeck effect *vs.* chemical potential (*µ* (eV)) in the range of −2 eV to 3 eV. In the valence band, the *S* is zero for AlMgCl_3_, but for AlMgI_3_, the *S* is positive around −1.3 eV, showing the dominance of hole carriers, and it becomes negative around −1.2 eV, suggesting the dominance of the electronic concentration. In the n-type region, the *S* is positive between 0 eV and 1 eV, showing the dominance of hole carriers, and is negative from 1.6 eV to 2.4 eV, which is an indication of electron concentration dominance in AlMgCl_3_. By contrast, for AlMgI_3_, the *S* is negative, showing that the electronic carrier concentration is dominant near the Fermi level. The maximum value of *S* in the n-type region for AlMgCl_3_ is found to be 3000 µV K^−1^ at around 0.7 eV, and for AlMgI_3_, it is found to be around −2900 µV K^−1^ at around 2.7 eV. Fig. 8(d) shows the power factor (*σS*^2^), which is a measure of the thermal performance of a material^100^ (Peltier effect, *i.e.* its efficiency to convert thermal energy into electrical energy and *vice versa*). The resulting values were plotted against the chemical potential, *µ*, in the range from −0.1 eV to 0.4 eV. The optimal values of the power factor for AlMgCl_3_ and AlMgI_3_ in the p-type region are found to be 4.5 × 10^11^ W m^−1^ K^−2^ and 7.6 × 10^11^ W m^−1^ K^−2^ at −0.07 and −0.01 eV, respectively. By contrast, in the n-type region, its maximum values for the materials are 8.1 × 10^11^ W m^−1^ K^−2^ at 0.2 eV for AlMgCl_3_ and 7.9 × 10^11^ W m^−1^ K^−2^ for AlMgI_3_ at 0.3 eV and 0.38 eV, respectively. Fig. 7(e) presents the figure of merit (*ZT*) *vs.* chemical potential in the range from −0.12 to 0.04 eV. On the negative potential side of the graph, *i.e.*, in the p-type region, its maximum values for AlMgCl_3_ and AlMgI_3_ are 0.48 and 0.2, respectively, at 0 eV and −0.01 eV, respectively, which suggests that the materials have p-type favourability. However, in the n-type region, AlMgI_3_ has no value of *ZT*, while AlMgCl_3_ has the maximum value of *ZT*, *i.e.*, 1 at 0.02 eV and above, which shows its suitability for thermoelectric devices.

**Fig. 9:**
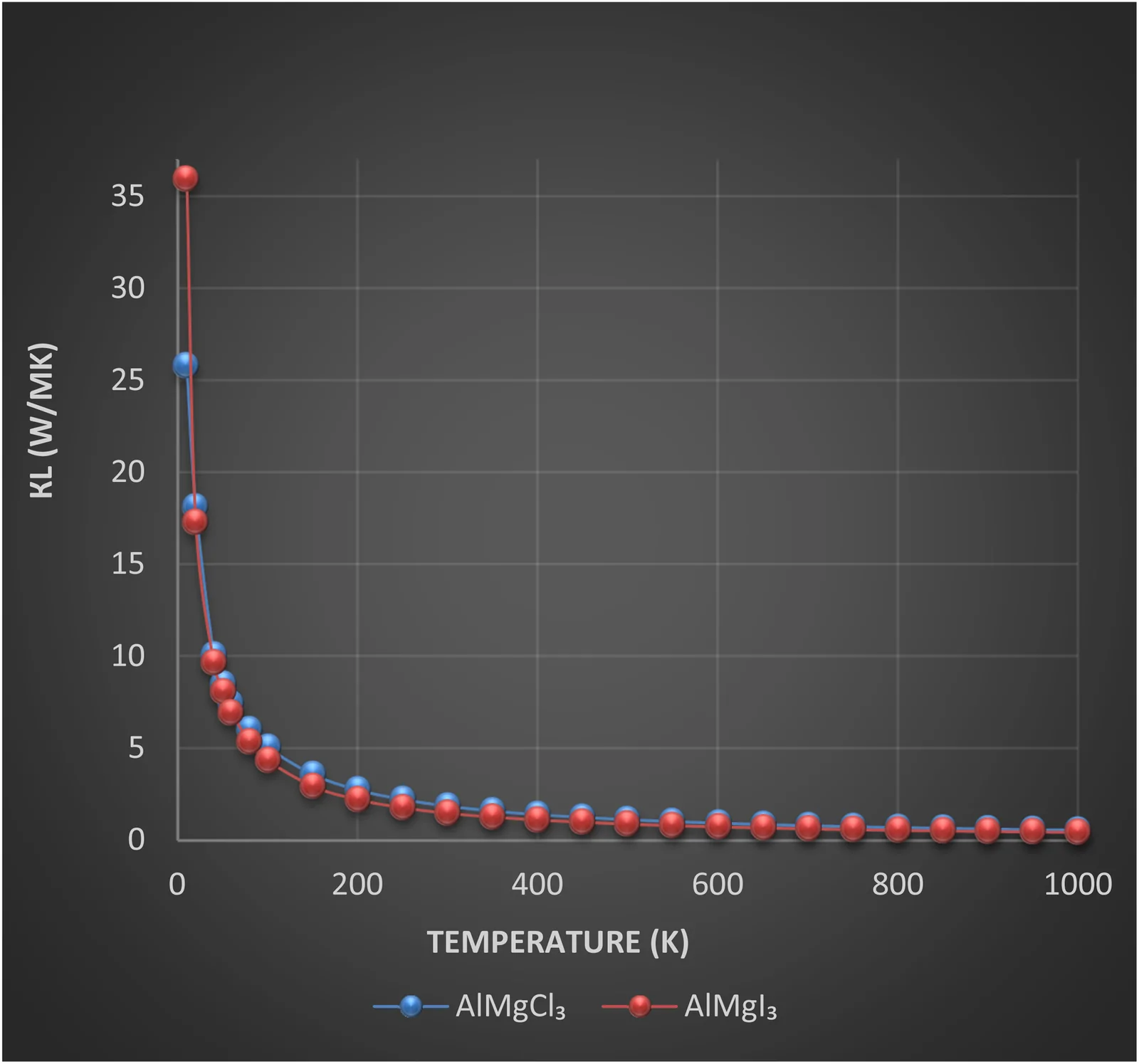
Computed lattice thermal conductivities of AlMgCl_3_ and AlMgI_3_.

Lattice thermal conductivity (*κl*) is a part of the total thermal conductivity that arises from the transport of heat through lattice vibrations. In the current study, the Phono3py^59^ code was employed to compute the lattice thermal conductivities (*κl*) of AlMgCl_3_ and AlMgI_3_ compounds. We determined *κl* according to the method reported in ref. 101 and 102. Fig. 9 shows the response of *κl* as a function of temperature (0–1000 K). Both AlMgCl_3_ (25.80 W mK^−1^) and AlMgI_3_ (35.94 W mK^−1^) have very high values of lattice thermal conductivity at a temperature of 10 K. These high values of *κl* at very low temperatures are due to negligible phonon–phonon scattering, allowing efficient heat conduction. However, as the value of temperature increases from 10 K to 200 K, the lattice thermal conductivity of both the studied materials drops sharply due to significant phonon–phonon interactions. At 300 K, the calculated values of *κl* for AlMgCl_3_ and AlMgI_3_ are 1.87 W mKs^−1^ and 1.48 W mKs^−1^, respectively, which are higher than those of X_3_MgNa (1.22 W mK^−1^ to 0.26 W mK^−1^)^102^ and comparable with those of PCdNa (2.6 W mK^−1^). After 600 K, *κl* exhibits an almost constant and similar trend for AlMgCl_3_ and AlMgI_3_ because phonon scattering reaches a limit. Moreover, AlMgCl_3_ has slightly higher lattice thermal conductivity than AlMgI_3_ at all temperature values except 10 K, indicating that AlMgCl_3_ may have stronger phonon transport efficiency. The discrepancy between the trends of lattice thermal conductivity of both compounds may be due to their different electronic properties. Specifically, high lattice thermal conductivity at low temperatures makes AlMgCl_3_ and AlMgI_3_ potential candidates for better performance in power electronics and industrial thermal systems.^103,104^ However, materials (AlMgCl_3_ and AlMgI_3_) having low lattice thermal conductivity at high temperatures are considered best for high-temperature insulation, such as coatings for jet engines, spacecraft components and gas turbines, to prevent heat damage.^105,106^

**Fig. 10:**
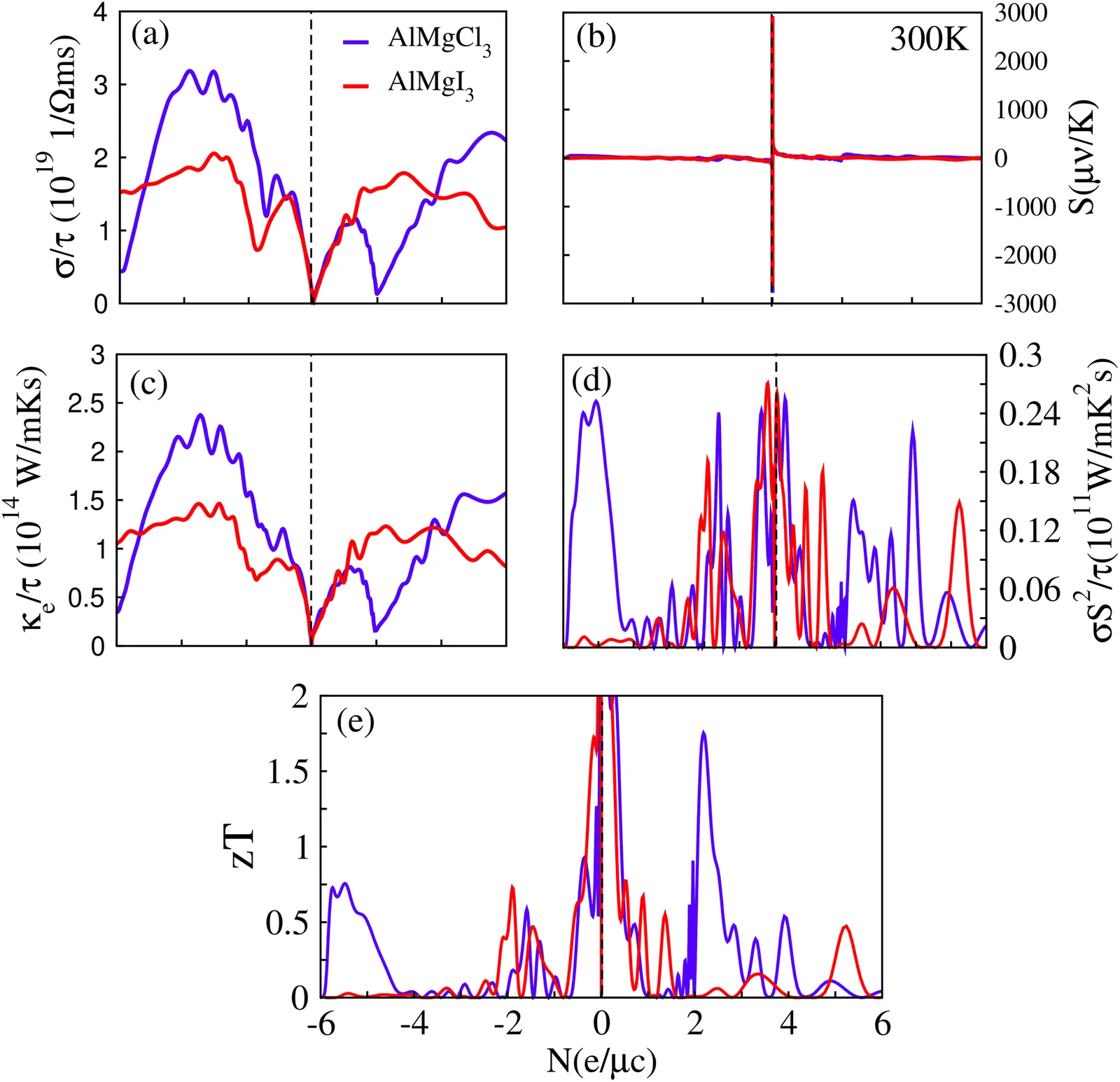
Calculated thermoelectric properties of AlMgX_3_ (X = Cl and I) *vs.* carrier concentration: (a) electrical conductivities (*σ*/*τ*), (b) Seebeck effect (*S*), (c) thermal conductivities (*κ*_e_/*τ*), (d) power factors (*σ*S^2^), and (e) figures of merit (*ZT*).

Fig. 10 shows the variation of the thermoelectric properties of AlMgX_3_ (X = Cl and I) against a change in the carrier concentration in the range from −6 eV to 6 eV. Fig. 10(a) depicts the change in the electrical conductivity (*σ*/*τ*) of the said materials in response to carrier concentration change within the set range. The *σ*/*τ* ratio reaches a peak value of 3.2 × 10^19^ (Ω ms)^−1^ at – 3.8 eV for p-type AlMgCl_3_, whereas for AlMgI_3_, this value is found to be 2.2 × 10^19^ (Ω ms)^−1^ at −2.8 eV, showing that these two structures have the ability to supply holes for thermoelectric conductivity. The electron carrier concentrations of these two structures' N-type semiconducting behaviour are found to be 2.5 × 10^19^ (Ω ms)^−1^ and 1.8 × 10^19^ (Ω ms)^−1^, respectively, at around 5.5 eV and 2.8 eV, respectively, for AlMgCl_3_ and AlMgI_3_. At around 0 eV, the carrier concentrations for both the materials in p-type and n-type regions are found to be negligibly small. Fig. 10(b) shows the change in Seebeck effect (*S*(µV K^−1^)) with respect to the carrier concentration (*n*) in the energy range of −6 eV to 6 eV. The maximum value of *S* is found to be around 3000 µV K^−1^ for AlMgCl_3_ around the *E*_f_, *i.e.*, 0 eV. Its value decreases sharply in the n-type region and almost becomes zero in the range from 0 to 6 eV. By contrast, the contribution in the p-type region is almost nil in the energy range from −6 eV to 0 eV.

For AlMgI_3_, it is found to be around 100 µV K^−1^ at almost 2.3 eV in the n-type region and around 50 µV K^−1^ in the p-type region near −5.6 eV. Fig. 10(c) demonstrates the thermal conductivity (*κ*_e_/*τ*) of AlMgX_3_ (X = Cl and I) *vs.* carrier concentration in the energy range stated earlier. The maximum thermal conductivity for AlMgCl_3_, found around −3.2 eV, is approximately 2.4 × 10^14^ W m^−1^ K^−1^ s^−1^, while for AlMgI_3_, it is found to be approximately 1.5 × 10^14^ W m^−1^ K^−1^ s^−1^ at approximately −3.5 eV in the p-type region for hole concentration. The thermal conductivity (*κ*_e_/*τ*) of both the structures decreases with increasing energy until it becomes zero around 0 eV. In the n-type region, the peak value of this parameter for both the structures increases till 1 eV and then decreases. However, for AlMgCl_3_, the value of thermal conductivity fluctuates till 6 eV, and its maximum value is found to be 1.3 × 10^14^ W m^−1^ K^−1^ s^−1^ at around 1.5 eV, and for AlMgI_3_, its value sharply falls at around 1.8 eV and then rises till it becomes the maximum at 4.5 eV, which is found to be 1.8 × 10^14^ W m^−1^ K^−1^ s^−1^. Fig. 10(d) demonstrates the power factor of structures explored in response to the carrier concentration. The optimal values of the power factor for AlMgCl_3_ and AlMgI_3_ are found around −0.3 eV and −4.4 eV, which are estimated to be 0.27 × 10^11^ W m^−1^ K^−2^ s^−1^ and 0.25 × 10^11^ W m^−1^ K^−2^ s^−1^, respectively, in the p-type region and become zero around −1 eV and −1.5 eV, respectively, and then fluctuate with a change in energy. By contrast, in the n-type region, the PF values are noticed to be approximately 0.26 × 10^11^ W m^−1^ K^−2^ s^−1^ at 0.2 eV for AlMgCl_3_, and for AlMgI_3_, the greatest value of power factor is observed to be 0.25 × 10^11^ W m^−1^ K^−2^ s^−1^ around 0.5 eV, becomes 0 at about 1.2 eV and 1 eV and then fluctuates with a change in energy. Fig. 10(e) presents the *ZT* values of both the materials against N (e per µc), which shows that the *ZT* values of AlMgCl_3_ and AlMgI_3_ are around 0.18 and 0.16 at 0 eV, respectively. They decrease with increasing p-type doping, become zero at around −0.8 eV for both and then fluctuate with a change in energy. Similarly, the *ZT* value of AlMgI_3_ decreases, then becomes zero while fluctuating around 2 eV and then further fluctuates till 6 eV with an increase in N-type doping. By contrast, for AlMgI_3_, the *ZT* value increases with an increase in N-type doping, reaches 1.7 around 2.5 eV and then fluctuates until it becomes zero around 6 eV.

### 3.6 Photocatalytic properties

The photocatalytic behaviour of a compound depends on the charge carrier recombination rate, optimal band gap, and suitable redox potential. The ideal condition is achieved through a minimum recombination rate with stable charge carriers, a favourable redox potential, and a narrow energy gap value of the material irradiated under the visible spectrum. The redox potential of visible spectrum exposure can be found by aligning the VB maxima and the CB minima compared to the H_2_O reduction or oxidation potential. The locations of the VB maxima and the CB minima are predicted theoretically using the following relation:^55^*E*_CB_ = *χ* − *E*_elec_ − 0.5*E*_g_ and *E*_VB_ = *E*_CB_ + *E*_g_where *χ* represents the Mulliken electronegativity (ME), *E*_CB_ and *E*_VB_ show the VB maxima and the CB minima, respectively, and *E*_elec_ = −4.50 signifies the standard electrochemical potential with respect to the hydrogen reference scale. The ME is actually equal to the geometric mean of the constituent atoms, where the 1st ionization energy and electron affinity are effectively employed.

**Fig. 11:**
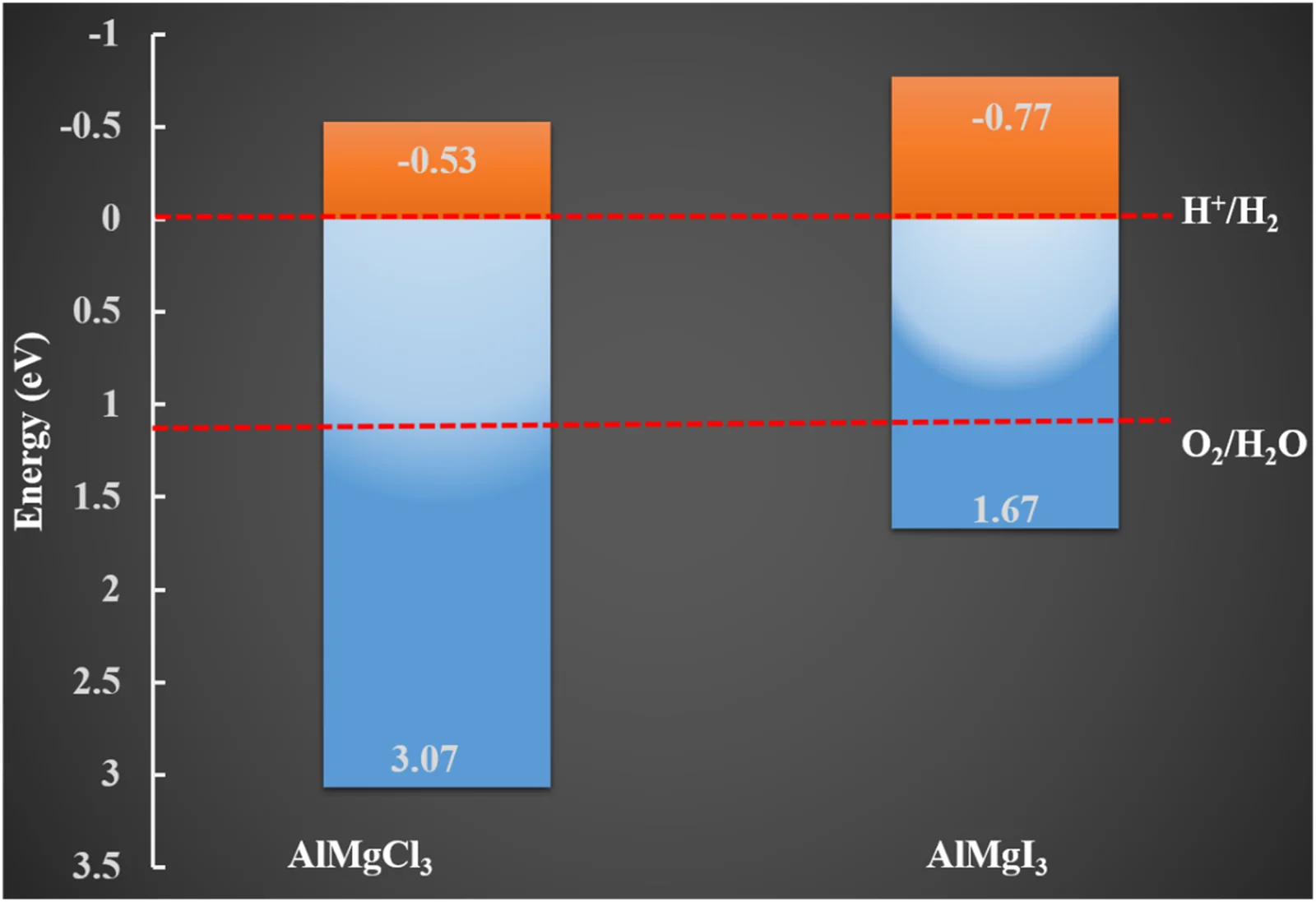
Calculation of VB and CB edge potentials of AlMgCl_3_ and AlMgI_3_.

The calculated MEs, *χ*, are 5.77 and 4.93, whereas the energy band gaps are found (Fig. 4) to be 3.60 and 2.44 for AlMgCl_3_ and AlMgI_3_, respectively. The VB minima potentials occur at 3.07 and 1.67, and the CB maxima potentials occur at −0.53 and −0.77 for AlMgCl_3_ and AlMgI_3_, respectively, as shown in Fig. 11. Through a comparison of these values, it can be seen that the VB minima responses can be ordered as AlMgCl_3_ > AlMgI_3_ with positive O_2_/H_2_O (1.23 eV), and the CB maxima can be ordered as AlMgCl_3_ < AlMgI_3_ to the H^+^ and H_2_ level. The findings of these analyses provide clear evidence that AlMgCl_3_ has the potential to oxidize water, from a thermodynamic perspective, producing O_2_; however, AlMgI_3_ is considered the best material to generate H_2_ due to its strong redox potential. Our computed results of Cs_2_PtCl_6_ for water splitting for O_2_ and H_2_ production are comparable with previously reported materials such as Mo-based Janus materials^107^ and CsPbX_3_ (X = Cl, Br, and I).^108^

### 3.7 Thermodynamic properties

**Fig. 12:**
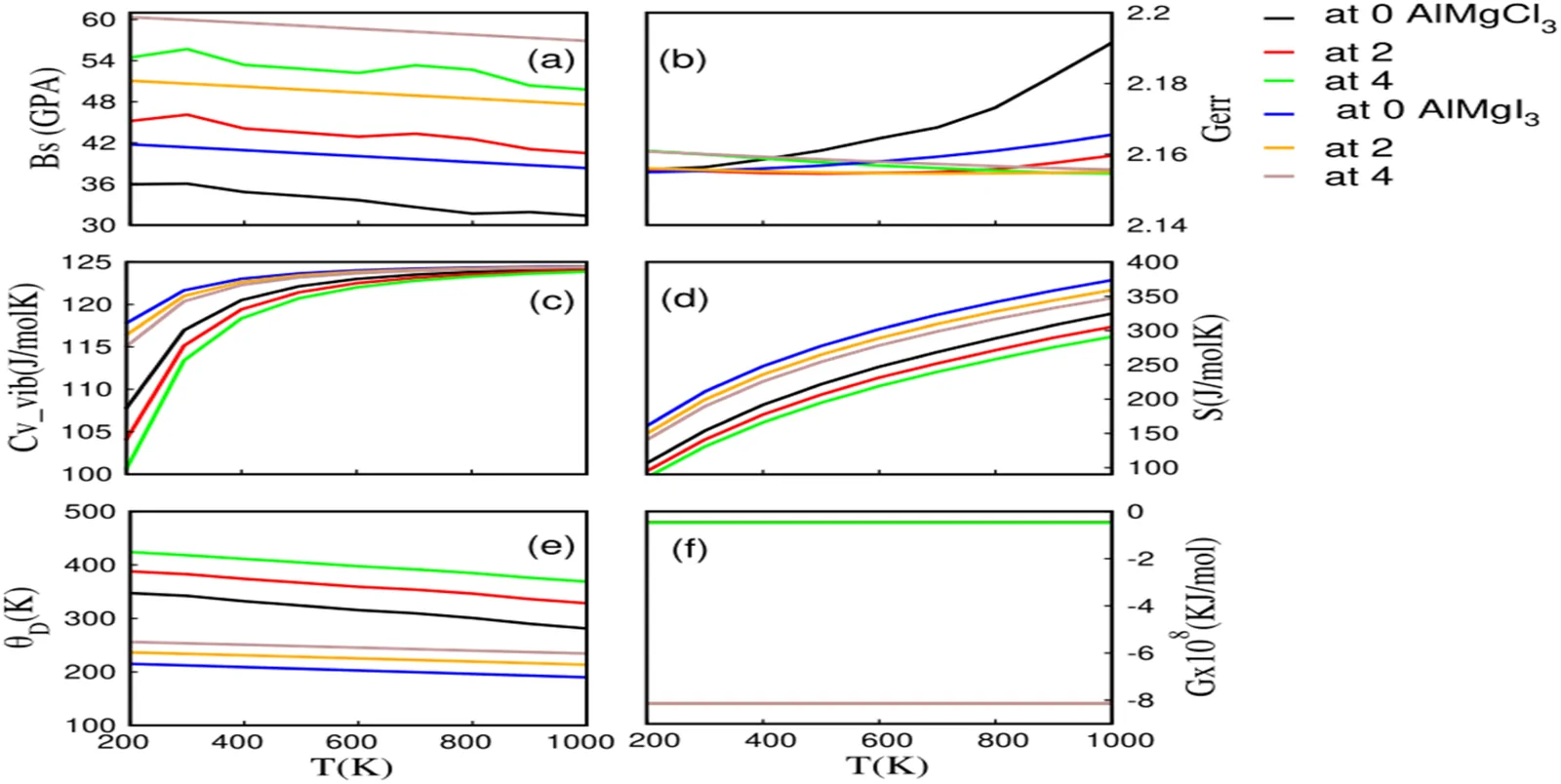
Calculated thermodynamic properties of AlMgX_3_ (X = Cl and I) *vs.* temperature (*T* (K)): (a) adiabatic bulk modulus (*B*_s_), (b) Helmholtz free energy (*G*_err_), (c) specific heat capacity (*C*_v_), (d) entropy (*S*), (e) Debye temperature (*θ*_D_), and (f) Gibbs free energy (*G*).

Studying thermodynamic properties is crucial for estimating the amount of heat required to vary the temperature of the material under investigation and the amount of work required to expand or compress the material.^65^ For this purpose, the quasi-harmonic Debye model was utilized within the 200 K to 1000 K temperature range and 0 to 4 GPa pressure range. We calculated the adiabatic bulk modulus (*B*_s_), Helmholtz free energy (*G*_err_), specific heat capacity (*C*_v_), entropy (*S*), Debye temperature (*θ*_D_), and Gibbs free energy (*G*) in the stated temperature and pressure ranges. The bulk modulus shows a material's opposition to the deformation produced under a constant heat content. The Debye temperature measures a material's intermolecular bond, whereas the Helmholtz free energy corresponds to a material's thermodynamic stability and the maximum amount of work done under a constant volume and temperature. Gibbs free energy is related to a material's stability with respect to temperature and pressure.^65,109–111^Fig. 12(a) shows the variation of the adiabatic bulk modulus (*B*_s_) of our material under pressure and temperature, which gives an insight into the material's mechanical properties. It is obvious from the figure that at a constant temperature, the value of the bulk modulus increases with increasing pressure, and hence, compressibility, which is the reciprocal of *B*_s_, decreases.^112^ Furthermore, the bulk modulus of AlMgCl_3_ changes with temperature, it increases from 200 K to 300 K, decreases from 300 K to 600 K and follows a nearly continuous downward trend from 600 K to 1000 K, whereas the bulk modulus of AlMgI_3_ decreases linearly from 200 K to 1000 K. The values of *B*_s_ for AlMgCl_3_ and AlMgI_3_ are 36 GPa and 41 GPa, respectively, at 200 K and 0 GPa. The maximum values of *B*_s_ for AlMgCl_3_ and AlMgI_3_ are 56 GPa and 60 GPa, found at 300 K and 200 K, respectively. Their values of *B*_s_ at 4 GPa and 1000 K are found to be 52 GPa and 58 GPa, respectively. Fig. 12(b) shows the variation of Helmholtz free energy in the temperature and pressure ranges mentioned in the graphs. At 0 GPa and 200 K, the values of the Helmholtz free energy of the two materials are the same, *i.e.*, approximately 2.155, which increases with temperature. AlMgCl_3_ shows a sharp increase in Helmholtz free energy compared to AlMgI_3_, and their values become approximately 2.186 and 2.164 at 1000 K, respectively. At 2 GPa, the *G*_err_ values of AlMgCl_3_ show a slightly increasing trend with an increase in temperature, from approximately 2.156 at 200 K to 2.166 at 1000 K, while for AlMgI_3_, the *G*_err_ values remain almost constant from 200 K to 1000 K. At 4 GPa, their *G*_err_ values show the same slightly decreasing trend from almost 2.16 at 200 K to 2.154 at 1000 K. Fig. 12(c) shows the variation of vibrational specific heat capacity (*C*_v_) *vs.* temperature *T* (K), which is the measure of the amount of energy needed to increase or decrease the temperature of a unit mass of a substance by 1 K, and the material's capability to store heat and qualifies its phase transition.^113^ It is obvious from the graph that at 200 K, the specific heat capacity decreases with an increase in pressure. Its values for AlMgCl_3_ and AlMgI_3_ are 108 J mol^−1^ K^−1^ and 118 J mol^−1^ K^−1^ at 0 GPa, respectively. Its values at 2 GPa and 4 GPa become 104 J mol^−1^ K^−1^, 102 J mol^−1^ K^−1^ and 116 J mol^−1^ K^−1^, 115 J mol^−1^ K^−1^ for AlMgCl_3_ and AlMgI_3_, respectively. The values of *C*_v_ at 300 K for AlMgCl_3_ are 117, 115 and 113 J mol^−1^ K^−1^ at 0 GPa, 2 GPa and 4 GPa, respectively. Similarly, the values of *C*_v_ at 300 K for AlMgI_3_ are 122, 121 and 120 J mol^−1^ K^−1^ at 0 GPa, 2 GPa and 4 GPa, respectively. The values of *C*_v_ for both the materials increase with increasing temperature until they become constant at around 800 K. Fig. 12(d) shows the effect of temperature on the change in entropy, which is a parameter used to study the structural, thermodynamic and magnetic stability of a material and indicates the phase change, lattice dynamics and disorder produced in the material with the variation of temperature.^114^ It is clear from the figure that the entropy increases with an increase in temperature and pressure. At 200 K and 0 GPa, the entropy values of AlMgCl_3_ and AlMgI_3_ are 120 J mol^−1^ K^−1^ and 175 J mol^−1^ K^−1^, respectively. They decrease with an increase in pressure and become 100 J mol^−1^ K^−1^ and 90 J mol^−1^ K^−1^ at 2 GPa and 4 GPa for AlMgCl_3_ and 155 J mol^−1^ K^−1^ and 145 J mol^−1^ K^−1^ for AlMgI_3_, respectively. They increase with an increase in temperature and pressure till they become 170 J mol^−1^ K^−1^, 150 J mol^−1^ K^−1^ and 140 J mol^−1^ K^−1^ at 0 GPa, 2 GPa and 4 GPa, respectively, for AlMgCl_3_ and 225 J mol^−1^ K^−1^, 210 K and 195 K at 300 K. They further increase to 320 J mol^−1^ K^−1^ and 375 J mol^−1^ K^−1^ at 1000 K and 0 GPa for AlMgCl_3_ and AlMgI_3_, respectively. The values at 2 GPa and 1000 K are 310 J mol^−1^ K^−1^ and 360 J mol^−1^ K^−1^, respectively, whereas at 4 GPa, the values are 290 J mol^−1^ K^−1^ and 350 J mol^−1^ K^−1^, respectively. Fig. 12(e) presents the variation of the Debye temperature with temperature. It shows the strength of the bond that exists between the molecules of a structure.^109^ Its values for AlMgCl_3_ and AlMgI_3_ are 212 K and 350 K, respectively, at 200 K and 0 GPa. Their values increase with an increase in pressure, which shows that the pressure causes the constituent materials to bind strongly, reducing the distance between their atoms. The values at 200 K and 2 GPa and 4 GPa are 230 K, 250 K and 395 K, 425 K for AlMgCl_3_ and AlMgI_3_, respectively. At room temperature (300 K), their values at 0 GPa, 2 GPa and 4 GPa are 205 K, 220 K and 245 K and 340 K, 380 K and 410 K for AlMgCl_3_ and AlMgI_3_, respectively. Their values decrease linearly at 1000 K, whereas at 0 GPa, 2 GPa and 4 GPa, they become 200 K, 215 K and 245 K, respectively, for AlMgCl_3_ and become 300 K, 350 K and 400 K, respectively, for AlMgI_3_. Fig. 12(f) shows the Gibbs free energy (*G*) variation with temperature. It is the parameter that represents the stability of a material with respect to temperature and pressure.^111^ This figure shows that there is no effect of variations in temperature and pressure on the Gibbs free energy, *i.e.*, it remains constant in the temperature range from 200 K to 1000 K and at pressures of 0 GPa, 2 GPa and 4 GPa. Its values for AlMgCl_3_ and AlMgI_3_ remain −0.4 and −8 kJ mol^−1^, respectively.

## 4 Conclusion

In this study, we employed first-principles density functional theory (DFT) to investigate the structural stability and optoelectronic and thermoelectric properties of lead-free perovskite halides, AlMgX_3_ (X = Cl and I), for renewable energy applications. We observed that AlMgX_3_ are energetically, mechanically, and thermodynamically stable due to low formation energies (−4.17 eV per f.u.), high *B*/*G* (2.13 and 2.97), and low fluctuations in the energies, according to MD simulations, respectively. Additionally, AlMgI_3_ displays enhanced ductility compared to AlMgCl_3_, attributed to weaker bond strength. Moreover, both materials exhibit a semiconducting nature. Interestingly, AlMgCl_3_ possesses a direct bandgap of 3.60 eV, which could be promising for optical devices due to low energy loss during the transition of electrons. Furthermore, the higher dielectric constants and sharp absorption peaks make them promising candidates for photovoltaic applications. Interestingly, the suitable band edges make them suitable for photocatalytic water splitting. Also, the thermoelectric performance evaluations demonstrate competitive figures of merit (*ZT*) of 0.54 and 0.61 for AlMgCl_3_ and AlMgI_3_, respectively, at room temperature. The latter's superior *ZT* stems from its lower bandgap and optimized carrier mobility. Lattice thermal conductivity decreases significantly at elevated temperatures, suggesting utility in high-temperature thermoelectric generators and heat management systems. Based on our results, the AlMgX_3_ perovskites could be viable alternatives to toxic lead-based perovskites due to their extraordinary properties. Future experimental synthesis and device integration are encouraged to validate these theoretical insights, paving the way for sustainable energy solutions in photovoltaics, optoelectronics, and waste-heat recovery technologies.

## Conflicts of interest

The authors declare that they have no known competing financial interests or personal relationships that could have appeared to influence the work reported in this paper.

## Data Availability

Data used in this research work will be made available from the authors on request.
